# Rational Design of Metal–Organic Frameworks for Pancreatic Cancer Therapy: from Machine Learning Screening to In Vivo Efficacy

**DOI:** 10.1002/adma.202412757

**Published:** 2025-02-02

**Authors:** Francesca Melle, Dhruv Menon, João Conniot, Jon Ostolaza‐Paraiso, Sergio Mercado, Jhenifer Oliveira, Xu Chen, Bárbara B. Mendes, João Conde, David Fairen‐Jimenez

**Affiliations:** ^1^ The Adsorption & Advanced Materials Laboratory (AAML) Department of Chemical Engineering & Biotechnology University of Cambridge Philippa Fawcett Drive Cambridge CB3 0AS UK; ^2^ NOVA Medical School Faculdade de Ciências Médicas NMS FCM Universidade NOVA de Lisboa Lisbon Portugal; ^3^ Comprehensive Health Research Centre (CHRC) NOVA Medical School Faculdade de Ciências Médicas NMS FCM Universidade NOVA de Lisboa Lisbon Portugal

**Keywords:** drug delivery, machine learning, metal–organic frameworks, pancreatic cancer, porous materials

## Abstract

Despite improvements in cancer survival rates, metastatic and surgery‐resistant cancers, such as pancreatic cancer, remain challenging, with poor prognoses and limited treatment options. Enhancing drug bioavailability in tumors, while minimizing off‐target effects, is crucial. Metal–organic frameworks (MOFs) have emerged as promising drug delivery vehicles owing to their high loading capacity, biocompatibility, and functional tunability. However, the vast chemical diversity of MOFs complicates the rational design of biocompatible materials. This study employed machine learning and molecular simulations to identify MOFs suitable for encapsulating gemcitabine, paclitaxel, and SN‐38, and identified PCN‐222 as an optimal candidate. Following drug loading, MOF formulations are improved for colloidal stability and biocompatibility. In vitro studies on pancreatic cancer cell lines have shown high biocompatibility, cellular internalization, and delayed drug release. Long‐term stability tests demonstrated a consistent performance over 12 months. In vivo studies in pancreatic tumor‐bearing mice revealed that paclitaxel‐loaded PCN‐222, particularly with a hydrogel for local administration, significantly reduced metastatic spread and tumor growth compared to the free drug. These findings underscore the potential of PCN‐222 as an effective drug delivery system for the treatment of hard‐to‐treat cancers.

## Graphical abstract

This work explores the MOF landscape to select a single, optimal candidate for successfully delivering cancer drugs (gemcitabine, paclitaxel, SN‐38) into tough pancreatic tumors. Machine learning and simulations guide this search, demonstrating colloidal stability, excellent biocompatibility, cellular uptake, and sustained release. With long‐term stability, the paclitaxel‐loaded hydrogel formulation achieves remarkable in vivo results, shrinking tumors and reducing metastasis.

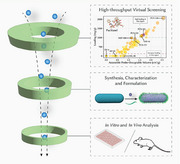

## 1 Introduction

Cancer remains the second leading cause of mortality worldwide, responsible for ≈10 million deaths annually — a number expected to rise to over 16 million by 2040.^[^ ^1^ ^]^ Despite advancements in cancer treatments and improved survival rates in recent years, the heterogeneous nature of cancer demands the development of new therapeutic strategies. This is particularly relevant for the so‐called “hard‐to‐treat” cancers, which continue to exhibit alarmingly high mortality rates with little to no progress in outcomes over the past 20 years.^[^ ^2^ ^]^ An example is pancreatic ductal adenocarcinoma (PDAC), the most common and deadly form of pancreatic cancer known for its exceptionally high mortality rate.^[^ ^2^, ^3^ ^]^ Despite accounting for only 3.3% of all new cancer cases in the United States, pancreatic cancer ranks as the fourth leading cause of cancer‐related deaths in males and females, with almost 7% of all cancer deaths.^[^ ^4^, ^5^ ^]^ The 5‐year relative survival rate for pancreatic cancer remains below 10%, starkly contrasting the higher survival rates seen in other common cancers such as breast (90.8%), prostate (97.1%), and thyroid (98.5%).^[^ ^6^ ^]^ This disparity highlights the aggressive nature of pancreatic cancer and the significant challenges it poses in early diagnosis and effective treatment. Pancreatic cancer is often diagnosed in later stages, which significantly limits treatment options. Only a small percentage of patients (15‐20%) are eligible for surgery, needing to rely on combination chemotherapy as the primary therapeutic approach.^[^ ^7^ ^]^ The first‐line treatment involves a combination of gemcitabine and albumin‐bound nanoparticle formulation of paclitaxel (nab‐paclitaxel) or FOLFIRINOX, which includes 5‐fluorouracil (5‐FU), leucovorin, irinotecan, and oxaliplatin.^[^ ^8^, ^9^ ^]^ Patients with disease progression after initial treatment may transition to a second‐line therapy, which typically includes a liposomal SN‐38 formulation combined with 5‐FU.^[^ ^10^ ^]^ However, these treatments come with notable limitations: gemcitabine is often limited by chemoresistance and systemic toxicity;^[^ ^11^, ^12^ ^]^ paclitaxel, while potent, faces challenges related to solubility and stability;^[^ ^13^ ^]^ and SN‐38, the active metabolite of irinotecan, despite its high potency, is highly hydrophobic and has limited solubility, which complicates its delivery and efficacy.^[^ ^14^ ^]^ This highlights the need for novel delivery systems that can enhance the effectiveness of chemotherapeutics while specifically targeting tumor cells.^[^ ^15^, ^16^, ^17^, ^18^ ^]^

Nanoparticle‐based delivery systems promote therapeutic effectiveness by improving tumor penetration, increasing cellular uptake, and reducing side effects, thereby optimizing the pharmacokinetics and biodistribution of therapeutic agents.^[^ ^19^ ^]^ Among these systems, metal‐organic frameworks (MOFs) have emerged as promising drug‐delivery materials.^[^ ^20^, ^21^ ^]^ MOFs consist of metal ions or clusters connected by organic ligands, offering a high chemical diversity and thousands of possible structures, as reported by the Cambridge Structural Database.^[^ ^22^, ^23^ ^]^ As a virtue of their self‐assembly, MOFs offer great control over their void space, allowing for the encapsulation of high quantities of active substances – often 20–50 times higher than their organic counterparts.^[^ ^24^ ^]^ Seminal work by Horcajada, Serre, et al.^[^ ^25^, ^26^ ^]^ introduced the concept of using MOFs for drug encapsulation and delivery, showing remarkable uptake of Ibuprofen in chromium‐based MIL‐100 and MIL‐101, and later iron‐carboxylate based flexible MOFs, notably MIL‐88. Morris et al.^[^ ^27^ ^]^ proposed the use of MOFs for the delivery of nitric oxide for applications pertaining to anti‐thrombosis, wound healing, and vasodilation. Lin et al.^[^ ^28^ ^]^ demonstrated the use of nanoscale MOFs for bioimaging applications. In 2010, Gref et al.^[^ ^29^ ^]^ reported the performance of these materials in vivo, thereby setting the stage for subsequent research. Since their initial introduction as drug delivery systems,^[^ ^20^, ^26^ ^]^ we have explored the design of MOF nanocarriers for the delivery of small molecule chemotherapeutic drugs,^[^ ^30^, ^31^, ^32^ ^]^ and small interfering RNA (siRNA).^[^ ^33^ ^]^ We have also studied and developed external surface modifications to target specific cells,^[^ ^34^ ^]^ and tune the cargo release at a controlled rate, thereby prolonging the effectiveness of the treatment and reducing side effects.^[^ ^35^, ^36^ ^]^

In the last few years, we have developed the use of zirconium‐based MOFs as drug delivery vehicles, achieving targeted delivery to both the cytosol^[^ ^35^ ^]^ and mitochondria.^[^ ^37^ ^]^ We have shown how the particle size and the surface chemistry of MOFs impact cellular uptake via endocytosis.^[^ ^31^, ^38^, ^39^ ^]^ Indeed, the characteristics of the nanoparticles affect cell interaction in vitro and in vivo.^[^ ^34^ ^]^ Given MOFs' loading and functionalization possibilities, their Achilles’ heel is arguably their lack of stability in the presence of phosphate salts typically found in natural environments. Recently, we have used this potential challenge as an opportunity to decorate and engineer the external surface of MOF nanoparticles.^[^ ^30^, ^31^ ^]^ First, using a methoxy‐PEG phosphate coating, we demonstrated improved chemical and colloidal stability and long‐term dispersity in vitro.^[^ ^31^ ^]^ Initially developed for PCN‐222, our PEGylation approach could also be extended to other nanosized zirconium MOFs such as UiO‐66, MOF‐808, NU‐901, and PCN‐128 – forming a mild and general formulation strategy.^[^ ^31^ ^]^ Second, we used a bilayer approach to expand the modulation of the external surface, avoid stability issues, and slow down drug release from typical 48 h in naked MOFs to 10+ days in decorated ones.^[^ ^30^ ^]^ In instances where post‐synthetic modification is not possible – for, e.g., for the delivery of large biomaterials such as proteins and viruses – Gassensmith et al. have reported biomimetic mineralization strategies for the development of MOF “depots” for extended drug release in vivo.^[^ ^40^ ^]^

Once one is able to engineer the external surface of a MOF, the challenge of developing them as drug delivery vehicles lies in their hybrid inorganic‐organic nature and the almost limitless chemical landscape.^[^ ^41^ ^]^ With over 115000 experimentally reported structures, selecting an optimal candidate becomes practically intractable if one relies solely on experimental approaches.^[^ ^42^ ^]^ Traditionally, researchers have relied on experimental expertise and chemical intuition to discover and design drug delivery vehicles. Over the years, this approach has yielded materials with impressive in vivo performances. However, the design space explored remains limited. Considering the significant translational gap between the laboratory and the clinic, as evidenced by the high attrition rates for clinical trials, there is a need to accelerate experimental timescales and explore a larger design space of materials.^[^ ^19^, ^43^ ^]^ In this context, efficiently utilizing computational techniques can disrupt current timescales.^[^ ^19^ ^]^

Here, we report an integrative approach that incorporates artificial intelligence/machine learning (AI/ML), computational chemistry, material synthesis, and formulation, with in vitro and in vivo testing to identify the most suitable MOFs for the delivery of chemotherapeutic agents, such as gemcitabine, paclitaxel, and SN‐38 (the active metabolite of irinotecan). First, we used AI/ML to shortlist biocompatible MOFs, drastically reducing the number of potential materials. Second, we used advanced computational techniques to predict and evaluate the encapsulation of the three chemotherapeutics within the biocompatible MOFs. This approach guided the selection of the most effective drug‐MOF combinations for subsequent experimental validation. Following computational screening, we experimentally loaded gemcitabine, paclitaxel, and SN‐38 into PCN‐222. This MOF was selected based on its experimental characteristics and potential performance. We then studied the performance of drug‐loaded PCN‐222 in vitro on a panel of pancreatic cancer cells (BxPC‐3, MIA‐PaCa‐2, and PANC‐1), followed by in vivo testing via intravenous (i.v.) and intraperitoneal (i.p.) routes. In both routes, we used a polyethylene glycol (PEG) coating on the external surface of PCN‐222, following our recent advances in the formulation of MOFs.^[^ ^31^ ^]^ In the case of i.p. local delivery, the MOF was combined with hyaluronic acid (HA)‐based hydrogels to maintain prolonged contact with abdominal organs. Our work proposes a transferable, hierarchical analysis of MOF‐based drug delivery systems to accelerate their selection and advance their formulation and translation to the clinic.

## 2 High‐Throughput Virtual Screening of MOFs

**Figure 1:**
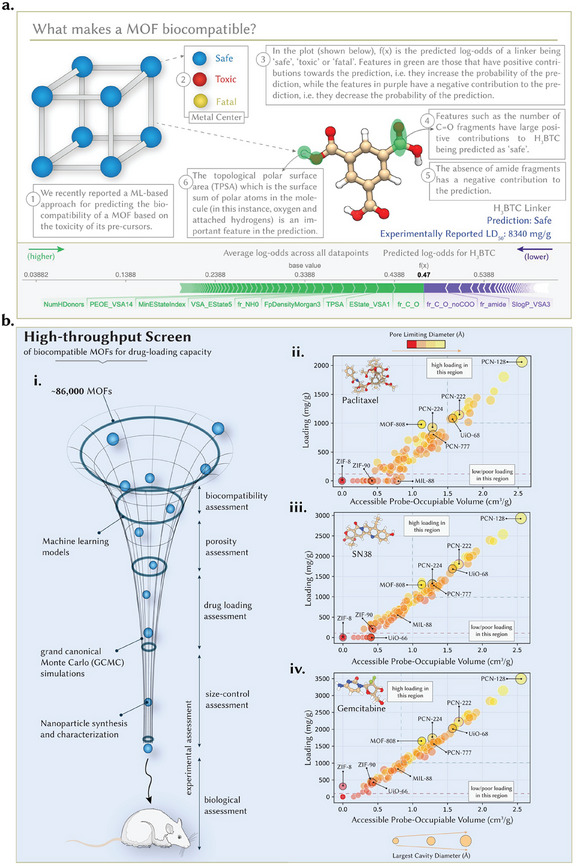
HTS of MOFs for the selection of candidates for drug delivery. a) Schematic illustration of previously reported ML methods for predicting the biocompatibility of a MOF based on the toxicity of its building blocks – the metal center and organic node. For the metal center, we curated a comprehensive database of toxicity profiles, while for the linker, we developed classifier algorithms that can predict a molecule to be “safe,” “toxic”, or “fatal.” The developed models are fully interpretable at the global and local levels. We employed the SHAP analysis at the local level to understand the predicted toxicity profile. As a representative example, we show the SHAP analysis of the widely used Benzene‐1,3,5‐tricarboxylic acid linker (H_3_BTC), which has an experimentally reported median lethal dose (LD50) of 8300 mg/g when administered to rats orally and was predicted to be “safe”. SHAP analysis helps pinpoint molecular features with large positive and negative contributions to the prediction. b. i. Integrative computational and experimental workflow representation. We initially screened 86000 MOFs from the CSD for their inherent biocompatibility, followed by GCMC simulations to assess their loading capacities of biocompatible MOFs for paclitaxel, gemcitabine, and SN‐38. MOFs that showed ultrahigh uptake were then assessed for their biological stability, ease of surface functionalization, and nanoparticle size control, after which a suitable candidate was selected for in vitro and in vivo testing. Drug loading capacities (mg/g) of biocompatible MOFs for ii. Paclitaxel, iii. SN‐38 and iv. Gemcitabine. The color indicates the PLD of the MOF, whereas the size indicates the MOF's largest cavity diameter (LCD). In all three cases, above a certain threshold, the drug loading scales almost linearly with pore volume. The data supporting HTS are available in Supporting Information.

Although experimental approaches are largely derived from chemical intuition and have led to the realization of efficient MOFs for drug delivery,^[^ ^44^, ^45^ ^]^ the procedures involved are labor‐intensive, time‐consuming, and expensive. Given the high attrition rates of the United States Food and Drug Administration (US FDA),^[^ ^43^ ^]^ scaling these efforts to clinical translation is essential. However, the vast diversity of MOFs makes the chemical landscape too complex to navigate solely using experimental methods. The ability of molecular simulations and ML to accurately predict the properties and behavior of materials, including MOFs, has emerged as a disruptive instrument in expediting the search for promising leads^[^ ^46^, ^47^ ^]^ and has recently been used to accelerate the discovery of drug delivery systems.^[^ ^48^, ^49^ ^]^ Although these advances are generally employed in energy applications such as carbon capture^[^ ^50^ ^]^ and hydrogen storage,^[^ ^51^ ^]^ integrating computational approaches in the MOF discovery pipeline would also advance efforts for the optimum selection of candidate structures for drug delivery applications. To ensure the synthetic accessibility of the leads, we limited our search space to the MOF subset of the Cambridge Structural Database (CSD), which contains over 86000 non‐disordered MOFs with defined synthesis protocols. Nevertheless, this pool was too large to be exhaustively screened using molecular simulations and included toxic metals such as Cd and Ni. Hence, we developed a hierarchical workflow to systematically narrow down the potential candidates (**Figure** **1**b,i). First, we defined a natural criterion as the inherent MOF biocompatibility. Conventionally, biocompatibility has been identified in a series of in vitro cell culture studies followed by in vivo animal models. However, these approaches are difficult to scale. To expedite the decision about what is biocompatible and what is not, we recently reported ML methods that predict the biocompatibility of a MOF based on its building blocks, that is, the metallic center and organic linker.^[^ ^49^ ^]^ For the metal center, we curated a comprehensive database of toxicity profiles while, for the organic linker, we developed ML‐based classification algorithms based on the United Nations “Globally Harmonized System for the Classification and Labelling of Chemicals” (GHS). The UN GHS classifies chemicals into five categories (Category 1 – 5) based on the median lethal dosage (LD_50_) post oral administration. For a more robust and accurate classification, here, we defined three custom categories and trained classifiers that can classify molecules as either “fatal,” “toxic,” or “safe,” with an accuracy > 83% (see Section S1, Supporting Information for further details). Using this ML‐based screening method, we obtained 809 highly biocompatible MOFs. We define highly biocompatible MOFs as those with biocompatible metal centers and organic linkers. In the context of metal centers, commonly used MOFs for drug delivery have potentially safer alternatives; here, Zr stands out. The classifier was fully interpretable for organic linkers at both global and local levels. At the global level, this allows us to extract molecular features that are highly correlated with toxicity. These include the presence or absence of molecular fragments, partial charges, and geometries. At the local level, we employed Shapley Additive Explanations (SHAP)^[^ ^22^ ^]^ to explain the predicted toxicity profiles of individual molecules, which allowed us to devise strategies to improve the biocompatibility of the MOF (Figure 1a). Notably, the screening successfully validated rational MOF choices from chemical and experimental intuition – with MOFs such as UiO‐66, PCN‐222, and PCN‐128 – as highly biocompatible.

The 809 biocompatible MOFs were screened for drug‐loading capacities, that is, the maximum amount of drug encapsulated per unit of mass. However, many of these biocompatible MOFs were non‐porous and were eliminated from consideration.^[^ ^49^ ^]^ While the ML‐based screening identified highly biocompatible MOFs, in practice, MOFs outside of this list are frequently used for drug delivery and have demonstrated reasonable performance in vivo. These MOFs are predominantly based on Fe and Zn, notably – ZIF‐8,^[^ ^52^ ^]^ and MIL‐88(Fe).^[^ ^40^ ^]^ Based on this experimental evidence, we included some Fe‐ and Zn‐centered MOFs, bringing our biocompatible, porous candidates to 143 MOFs. Table S1 (Supporting Information) shows a list of MOFs, along with their calculated textural properties. We used grand canonical Monte Carlo (GCMC) simulations to calculate the drug‐loading capacities of these candidate structures (see Supporting Information S1 for full details). GCMC is a method rooted in statistical mechanics that is ideal for modeling adsorption phenomena in bulk systems as it is good at accounting for density fluctuations at a fixed volume and temperature^[^ ^53^ ^]^ which has demonstrated good performance when compared with experiments.^[^ ^54^ ^]^

Intuitively, the drug‐loading capacity of a MOF should be scaled linearly with pore volume. The larger the available void space, the larger the amount of drug that can be encapsulated. However, some aspects must be considered. For instance, in some cases, despite having relatively large pore volumes, the pore opening of the MOF, also known as the pore‐limiting diameter (PLD), is small, preventing a drug from entering and accessing the void space. In other cases, the chemistry of the MOF is incompatible with that of the guest such as when the MOF's internal porosity is hydrophobic, and the guest molecule is hydrophilic. To analyze drug uptake, we performed high‐throughput screening (HTS) to assess the ability of candidate biocompatible MOFs to encapsulate the agents used in the current standard of care for pancreatic cancer therapy on these 143 biocompatible MOFs exclusively.^[^ ^55^, ^56^, ^57^ ^]^ Figure 1b ii‐iv shows the loading capacity of gemcitabine, paclitaxel, and SN‐38 on the 143 structures with respect to the guest accessible pore volume of the candidate MOF structures; Table S1 (Supporting Information) shows a list of drug loading capacities of the candidate MOFs. In all three cases, a threshold generally corresponds to a specific pore size, where the drugs cannot accommodate the porosity given their dimensions. Above this threshold, drug uptake scales linearly with pore volume. In addition to pore volume, drug loading capacities vary with the size of the drug, with gemcitabine, the smaller drug, showing the highest average loading capacity, and paclitaxel, the larger one, showing the lowest. Indeed, the smaller the drug, the easier it is to accommodate and pack it in the porosity. At a threshold of 1 g of drug loading per g of MOF (that is, a loading capacity of 50 wt.%), we were left with a candidate set of ca. 15 MOFs that can accommodate appreciably high quantities of all three drugs. It is important to note that in experimental settings, it is anticipated that the loading could be lower owing to several assumptions involved in the GCMC simulations, such as vacuum conditions and the absence of any solvents. However, previous studies have shown that GCMC simulations with and without solvents offer similar results and can accurately predict experimental trends.^[^ ^54^ ^]^ In our case, commonly used MOFs such as ZIF‐8, ZIF‐90, and MIL‐88 showed poor drug loading capacities due to their narrow pore sizes, whereas MOFs such as PCN‐224 and MOF‐808 showed moderate uptake. In contrast, PCN‐222, UiO‐68, and PCN‐128 exhibited high uptakes owing to their large pore sizes and volumes.

## 3 Selecting Optimal MOFs by Stability, Modifiability, and Biomedical Suitability

**Figure 2:**
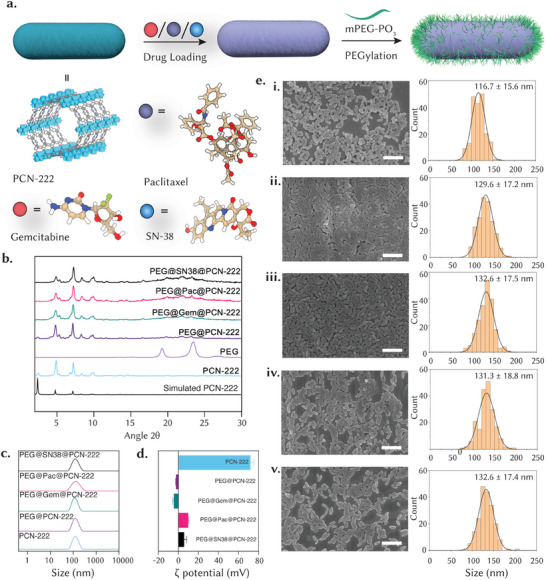
Characterization of drug‐loaded PEG@PCN‐222. a) Representation of the chemical structure of PCN‐222, gemcitabine, paclitaxel and SN‐38 together with a schematic illustration of the drug loading and PEGylation of PCN‐222. b) Simulated and experimental PXRD patterns. The loaded MOFs confirmed the characteristic peaks of simulated PCN‐222. c) Average diameters of PCN‐222 (blue line) PEG@PCN‐222 (purple line), and drug‐loaded PCN‐222 (green, pink, and black lines) in water (*n* = 3). d) Zeta potentials of water suspensions of PCN‐222, PEG@PCN‐222, and drug‐loaded PCN‐222. e) SEM images of i. PCN‐222, ii. PEG@PCN‐222, iii. PEG@Gem@PCN‐222, iv. PEG@Pac@PCN‐222, and v. PEG@SN38@PCN‐222. Particle sizes were calculated by measuring ≈200 particles using ImageJ software (scale bar = 500 nm).

Although biocompatibility and drug‐loading capacity are critical factors in the selection of MOFs for drug loading and delivery, other parameters are also important. In particular, an optimal MOF should present adequate stability in biological systems; therefore, it does not show bioaccumulation, and its external surface should be easily modified post‐synthetically so that different functionalities can be added.^[^ ^34^ ^]^ In addition, properties such as the fluorescent nature for easy tracking, synthetic reproducibility, and control over particle size are important characteristics.^[^ ^31^, ^42^, ^44^, ^49^ ^]^ While controlled drug release is another critical factor for drug delivery, most studies indicate that regardless of the MOF used, drug release is observed within 24 h, occasionally extending to 48 h.^[^ ^30^ ^]^ We would thus rely on our recent advances in the development of formulation strategies, notably, the application of polymer coatings (such as PEG) on the external MOF surface, which has emerged as a MOF‐agnostic strategy to achieve controlled release profiles.^[^ ^31^ ^]^ Based on our previous work and experience, we selected PCN‐222 for subsequent experimental testing and validation due to its high porosity, optimal stability^[^ ^31^, ^34^, ^44^, ^58^ ^]^ and the relative ease of synthesis and particle size control, despite earlier reports highlighting challenges in its synthesis.^[^ ^59^ ^]^ Other MOFs, such as PCN‐128, show superior uptake; however, nanoparticle synthesis is more challenging. PCN‐222 consists of Zr_6_ clusters with eight edges connected to the tetrakis (4‐carboxyphenyl) porphyrin (TCPP) linkers featuring 1D micro‐ (triangular) and mesoporous (hexagonal) channels with diameters of 1.7 and 3.6 nm, respectively. We have previously reported that the particle size of PCN‐222 could be finely controlled by varying the amount of modulator trifluoroacetic acid (TFA) during the solvothermal synthesis with TCPP and Zr_6_ clusters.^[^ ^31^ ^]^ Here, by optimizing the TFA content, we successfully produced PCN‐222 particles ≈120 nm in size, which is well suited for biomedical applications. **Table** **1** shows the average diameter of PCN‐222 using both scanning electron microscopy (SEM) and dynamic light scattering (DLS). DLS analysis indicated an average diameter of 123.4 nm with a polydispersity index of 0.11 (Figure 1c and Table 1) while the SEM analysis confirmed the ellipsoid‐shaped nanoparticles and size uniformity (**Figure** **2**e‐i). The z‐potential of PCN‐222 resulted in 71.9 mV (Figure 2d and Table 1), suggesting the predominance of positively charged end groups on the external surface of the nanoparticles. Additionally, powder X‐ray diffraction (PXRD) revealed the presence of peaks that matched the pattern predicted from the single‐crystal structure, confirming the phase purity of PCN‐222 (Figure 2b).

**Table 1:** Characterization of the PCN‐222‐based nanoparticles. Characterizations included particle size measured by SEM, effective diameter, and polydispersity index (PdI) measured by DLS, z‐potential, and drug loading (by UV–vis) of the different samples. SEM values represent the standard deviation of the 200 particles.

	Particle size [nm]^a)^	Effective diameter [nm]^b)^	PdI	Z‐potential [mV]	Drug loading [wt.%]
PCN‐222	116.7 ± 15.6	123.4 ± 1.3	0.11	71.9 ± 2.7	–
PEG@PCN‐222	129.6 ± 17.2	132.2 ± 0.3	0.13	−2.4 ± 0.3	–
PEG@Gem@PCN‐222	132.6 ± 17.5	134.2 ± 0.7	0.08	−4.6 ± 0.8	30.2
PEG@Pac@PCN‐222	131.3 ± 18.8	132.1 ± 1.3	0.14	9.7 ± 0.4	40.8
PEG@SN38@PCN‐222	132.6 ± 17.4	145.9 ± 0.8	0.18	5.7 ± 2.1	30.1

^a)^ Measured by SEM – errors represent the standard deviation of 200 particles;

^b)^ Measured by DLS in water.

Following the synthesis, we analyzed the experimental loading capacities for the three selected anticancer drugs, namely gemcitabine, paclitaxel, and SN‐38.^[^ ^8^, ^9^ ^]^ Each of these drugs has its own set of limitations when used in conventional formulations. Gemcitabine requires frequent administration because of its rapid metabolism and elimination; paclitaxel is poorly soluble in water, which complicates its delivery and can cause severe hypersensitivity reactions due to the solvents used in its formulation, and SN‐38 is also highly hydrophobic, limiting its bioavailability and therapeutic potential.^[^ ^14^, ^16^, ^60^ ^]^ Hence, gemcitabine, paclitaxel, and SN‐38 were dissolved in water, ethanol, and DMSO, respectively, and mixed with a corresponding solution of PCN‐222 (Figure 2a). After incubation for three days, the excess solution was removed, and the drug‐loaded MOFs were resuspended in an aqueous solution of mPEG‐PO_3_ for two days (Figure 2a), following our previous formulation protocols.^[^ ^31^ ^]^ Our previous studies on formulation with mPEG‐PO_3_ have not only shown controlled release profiles, but also improved biocompatibility when compared to the bare MOF, especially at higher concentrations.^[^ ^31^ ^]^ The phosphate head of PEG strongly binds the Zr clusters, providing the MOFs with an external PEG layer that allows for colloidal stability in biological media.^[^ ^31^ ^]^ Following drug loading and characterization, the final formulations, named PEG@drug@PCN‐222, were maintained in an aqueous suspension until further use. Using UV–vis spectroscopy on the mother solutions before and after drug loading (Figure S5, Supporting Information), we found drug uptakes of 30.2, 40.8, and 30.1 wt. % for gemcitabine, paclitaxel, and SN‐38, respectively. Figure 2e shows the SEM images of bare PCN‐222 and PEGylated PCN‐222 with and without loaded drugs. All systems preserved the typical ellipsoidal morphology of PCN‐222. DLS and SEM measurements showed an increase in the particle size of ca. 10 nm following PEGylation, consistent with previous studies,^[^ ^31^ ^]^ with DLS showing low polydispersity values, confirming good colloidal stability in water (Table 1). The z‐potential measurements showed a decrease in the surface charge post‐PEGylation, where the initial superficial charge dropped from 71.9 mV for PCN‐222 to ‐2.4 mV for PEG@PCN‐222 and ‐4.6, 9.7, 5.7 mV for gemcitabine, paclitaxel, and SN‐38 loaded PCN‐222, respectively (Figure 2d and Table 1). This change confirmed the presence of PEG on the external surface. The PXRD patterns confirmed the crystalline phase, where the first five peaks matched both the simulated and bare PCN‐222, confirming that crystallinity was maintained after loading and PEGylation. However, the PEG@drug@PCN‐222 samples showed differences, with the first peak (100) showing a decrease in intensity probably related to the presence of some mPEG‐PO_3_ molecules infiltrated into the mesoporous cavities. The samples were further characterized by thermogravimetric analysis (TGA) and Fourier transform infrared (FT‐IR) spectroscopy, as shown in Figures S6 and S7, confirming both the presence of drugs and PEG.^[^ ^31^ ^]^

## 4 Cytotoxicity and Cell Internalization of PEGylated PCN‐222

Pancreatic cancer, marked by its high morphological heterogeneity, presents a significant challenge in the development of effective therapeutic strategies.^[^ ^2^, ^4^, ^61^, ^62^ ^]^ This heterogeneity is reflected in the varying cellular compositions and architectures within the tumor microenvironment, significantly influencing the biological behavior of the tumor and its interaction with therapeutic agents.^[^ ^7^, ^63^ ^]^ As a result, this diversity leads to a poor and unpredictable response to conventional chemotherapeutic drugs, complicating treatment regimens and often leading to therapeutic resistance.^[^ ^60^ ^]^ This heterogeneity must be considered during the development of drug‐delivery systems. For instance, over 95% of pancreatic cancers carry KRAS^G12D^ mutations, and 70% carry TP53 mutations.^[^ ^62^ ^]^ In our study, we tested the in vitro toxicity and internalization of our systems on a panel of three different pancreatic cancer cell lines: BxPC‐3 (wt‐KRAS, TP53^Y220C^), MIA‐PaCa‐2 (KRAS^G12C^, TP53^R248W^), and PANC‐1 (KRAS^G12D^, TP53^R273H^).

**Figure 3:**
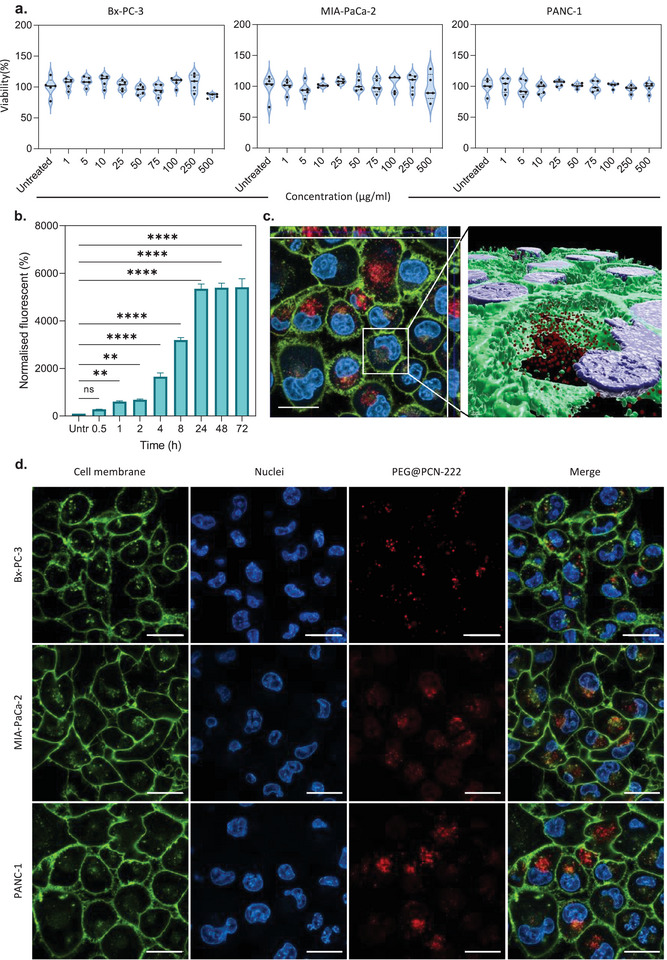
Biocompatibility and cellular uptake of PEG@PCN‐222 in PDAC cells. a) MTS assay of PEG@PCN‐222 in pancreatic cancer cells (BxPC‐3, MIA‐PaCa‐2, and PANC‐1) at different concentrations after 72‐h of incubation. b) Time‐point uptake of PEG@PCN‐222 in PANC‐1 cells using flow‐cytometry. c) 3D reconstruction of the z‐stack of PANC‐1 using the Imaris software. d) Confocal images of PDAC cells (BxPC‐3, MIA‐PaCa‐2, and PANC‐1) after 24 h incubation with 100 µg mL^−1^ PEG@PCN‐222. Cells were incubated with CellMask (green) and Hoechst 33342 (blue) to stain the cell membrane and nuclei, respectively. The red channel captures the fluorescence of PEG@PCN‐222. Scale bar 20 µm. Data are presented as mean ± SD (One‐way ANOVA *n* = 3, ****p<0.0001, ***p<0.001, **p<0.01, *p<0.05).

**Figure** **3**a shows the viability of cells treated with bare PEGylated PCN‐222 at 0–500 µg mL^−1^ concentrations using MTS endpoint assays to assess the material's toxicity. Figure S8 (Supporting Information) shows the cell growth monitored for 72 h using real‐time in vitro micro‐imaging on an Incucyte, a live cell analysis system that allows quantification of the confluence percentage as a function of time, a value directly linked to cell density. The main advantage of live‐imaging systems over standard endpoint assays, such as MTS, nuclei count, or CellTiter glow, is that they enable comparison between different time points and normalization of the data obtained in the same well over time. In addition, it allows for the quantification of cell surface area coverage as confluency values so that cell growth can be expressed as a ratio between the endpoint and time zero, eliminating possible errors in cell seeding and interactions of the MOFs with the colorimetric reagent.^[^ ^64^ ^]^ Both MTS assay (Figure 3a) and live cell analysis (Figure S8) confirmed that PEG@PCN‐222 showed no significant toxicity compared to untreated cells in all three pancreatic cancer cell lines. Figure 3b‐d shows the cellular internalization of PEG@PCN‐222 analyzed by flow cytometry and confocal microscopy, respectively. In particular, we exploited the fluorescent properties of the porphyrinic linker to detect PCN‐222 in the three cell lines. PEG@PCN‐222 was successfully internalized in all three cell lines, as highlighted by the 3D reconstruction confirming the localization of MOFs within the cytoplasm. The uptake of PEG@PCN‐222 increased with time in all PDAC cell lines for up to 24 h, reached saturation, and did not increase further at 48 and 72 h (Figure 3b).

## 5 Enhanced Efficacy of Drug‐Loaded MOF in Pancreatic Cancer Cell Growth Inhibition

**Figure 4:**
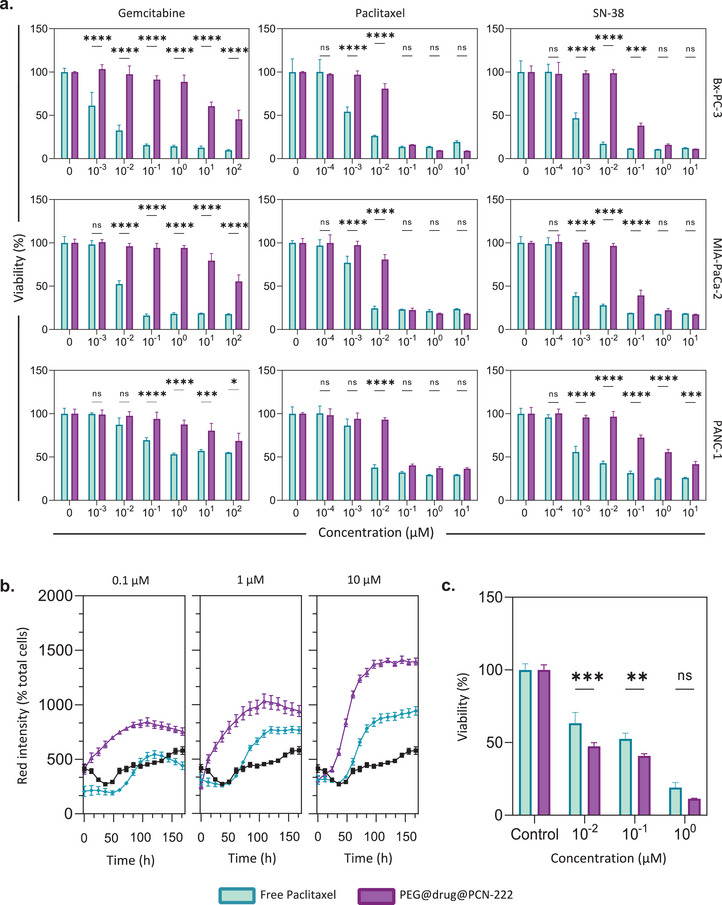
Cytotoxicity of PEG@drug@PCN‐222. a) MTS assay of PEG@Gem@PCN‐222, PEG@Pac@PCN‐222, and PEG@SN38@PCN‐222 at different concentrations after 72 h of incubation with different PDAC cell lines. The drug‐loaded MOFs formulations (purple) were compared with the same concentration of the free drug (blue) based on the drug‐loading values. b) Short‐incubation toxicity of PEG@Pac@PCN‐222 in PANC‐1 cells measured by live‐cell imaging. PANC‐1 cells were incubated for 6 h with different concentrations (left panel: 0.1 µg mL^−1^, the middle panel: 1 µg mL^−1^, and the right panel: 10 µg mL^−1^) of free paclitaxel (blue lines) or PEG@Pac@PCN‐222 (purple lines). The drug‐loaded MOFs formulations (purple) were compared with the same concentration of the free drug (blue) based on the drug‐loading values. c) MTS assay of short‐term incubation of PEG@Pac@PCN‐222 in PANC‐1 cells. The cells were incubated for 6 h with different concentrations of paclitaxel or PEG@Pac@PCN‐222. After incubation, the cells were washed twice with fresh medium and incubated with a medium containing CytotoxRed stain under normal growth conditions for 7 days. The cells were then tested using the MTS assay. Two‐way ANOVA statistical analysis was performed between the drug‐loaded MOFs and their free drug counterparts at a given concentration, as shown in the graphs. (*n* = 3; ns = non‐significant, ****p<0.0001, ***p<0.001, **p<0.01, *p<0.05).

After evaluating the tolerability and internalization of PEG@PCN‐222, we assessed its efficacy in inhibiting cancer cell growth compared to free drugs. It is important to note that all cell lines showed different sensitivities toward the standard‐of‐care drugs (Table S2, Supporting Information). This variability in drug response among cell lines makes them a suitable in vitro model for evaluating the drug delivery potential of nanocarriers. By comparing the efficacy of nanocarriers across these diverse cell lines, we can obtain a more comprehensive therapeutic potential and limitations.^[^ ^64^ ^]^ **Figure** **4**a shows the antiproliferative effects of free drugs and loaded ones to PEG@Gem@PCN‐222, PEG@Pac@PCN‐222, and PEG@SN38@PCN‐222 – in the three cell lines at endpoint, MTS assays. All cells were treated for 72 h with the same drug concentration as the free and PCN‐222‐loaded drugs. As expected, all systems showed concentration‐dependent decrease in viability. In addition to the endpoint MTS analysis, we calculated the proliferation IC50 values from the obtained dose response for both MTS and live cell analysis (Table S2 and Figures S9–S11, Supporting Information). The IC50 values for the free drug calculated from the live‐cell image analysis assay correspond to those reported in the literature,^[^ ^65^ ^]^ with drug‐loaded MOFs showing lower IC50 values for all tested cell lines. On the other hand, the IC50 values obtained from the MTS assay were 10‐fold lower than those obtained from the live‐cell image analysis for both gemcitabine and SN‐38 alone while presenting comparative values for the drug‐loaded MOFs (Figures S9 and S11, Supporting Information). This highlights the importance of using different methods to evaluate new therapies and avoid generating misleading results. Looking broadly at the MTS and live cell imaging results, high concentrations (>1 µM) of paclitaxel‐ and SN‐38‐loaded PEG@PCN‐222 had a similar effect to that of the free drugs. However, at concentrations lower than the IC50 values, all three drug‐loaded MOFs showed lower antiproliferative effects than the respective free drugs. We hypothesize that this lower cytotoxic behavior is due to the presence of PEG, which has a delayed‐release effect on the encapsulated drug, thus preventing burst release and allowing slow cargo release, reducing its overall, time‐dependent toxicity.^[^ ^31^ ^]^

Importantly, in a real in vivo experiment, a systemic drug delivery vehicle or a free drug will never be in contact with target cells for more than 24 hours. To replicate this during in vitro experiments, one needs to monitor the intake of the vehicle or free drug during the first hours. Here, we studied the slow drug release of drug‐loaded MOFs compared to free drugs by assessing PEG@Pac@PCN‐222 in vitro for a short‐term incubation response. Briefly, we incubated MOF‐loaded and free drugs in cell media with cells for 6 h. Following incubation, the cells were washed with media and incubated with a drug‐free medium for a week, with the medium replaced every three days. This allowed us to show the effects of drug release from the MOFs over time. Figure 4b shows the short‐incubation toxicity of PEG@Pac@PCN‐222 in PANC‐1 cells measured by live‐cell imaging; Figures S12 and S13 (Supporting Information) show the Incucyte images of PANC‐1 cells incubated for 6 h with paclitaxel and PEG@Pac@PCN‐222 on days 0 and 7 using the Incucyte Cytotox Red reagent, a nucleic acid dye that stains dead or dying cells. Free paclitaxel and PEG@Pac@PCN‐222 reduced cell viability in a time‐ and concentration‐dependent manner, corresponding to an increase in the red signal. Low concentrations of paclitaxel (0.1 µM) showed low toxicity compared to the untreated cells (b, left panel). In contrast, higher concentrations of PEG@Pac@PCN‐222 (Figure 4b, middle and right panel) showed more dead (red) cells than those incubated only with paclitaxel. After seven days, the cells were tested using an MTS assay (Figure 4c), confirming that, following the short‐term exposure of PANC‐1 cells to both free paclitaxel and PEG@Pac@PCN‐222, the loaded MOFs showed a higher antiproliferative effect than the same free drug concentration. Again, this strongly corroborates our hypothesis of the slow release of the drug from the MOFs through the PEG coating mechanism. After 6 h of incubation, part of the free drug and MOF was internalized in the cells, and the remaining materials were washed away. The fact that the MOF system is more cytotoxic means that a higher amount of drug has been internalized than the free drug. In other words, loading paclitaxel into PCN‐222 provides a sustained release of the cargo in vitro, reducing the burst release effect while enhancing the drug uptake in shorter times. PEG@Pac@PCN‐222 was further explored for in vivo experiments to assess the efficacy of the system in a physiological context.

## 6 Long‐Term Stability of PEG@Pac@PCN‐222

The long‐term stability of drug delivery systems represents a major drawback in translating promising formulations into the clinic.^[^ ^19^ ^]^ Ensuring that these nanoparticles retain their therapeutic properties over time is necessary to achieve reproducible outcomes in a clinical setting. If unstable, a nanoparticle system can degrade or prematurely release its payload, leading to diminished effectiveness and potentially adverse side effects. Stability is a prerequisite for regulatory bodies, which often requires comprehensive stability and shelf‐life data to confirm that a product will remain safe and effective throughout its intended shelf life.^[^ ^42^ ^]^ In this study, we assessed the stability of our formulated PEG@Pac@PCN‐222 in water for over one year. The samples were synthesized, loaded, and PEG‐formulated as previously described and kept in the dark at 4 °C. We then evaluated the colloidal stability of PEG@Pac@PCN‐222 using DLS. Figure S14a (Supporting Information) shows the hydrodynamic size of PEG@Pac@PCN‐222 over 12 months, with a consistent value of ≈130 nm and negligible variation over time. At 12 months, the z‐potential of PEG@Pac@PCN‐222 was 9.4 ± 0.7 mV, similar to the 9.7 ± 0.4 mV initial value at time zero. Both DLS and z‐potential showed that the formulation allowed for the colloidal stability of PEG@Pac@PCN‐222 at the storage conditions tested, showing the potential for long‐term storage. We also analyzed any possible toxic effects due to partial degradation of the free drug MOF. Figure S14c (Supporting Information) shows the MTS assay results of BxPC‐3 cells incubated with PEG@PCN‐222 at month zero and after storage at 4 °C for 12 months. The cell viability at month zero and after 12 months of storage did not show any differences, with no toxicity up to 200 µg mL^−1^. At 500 µg mL^−1^, a high relative concentration, the sample at 12 months showed higher toxicity (ca. 50% cell viability). This confirmed the high stability after one year with no significant toxic effects at clinically relevant concentrations. The formulation was then tested in vitro to confirm its unaltered toxicity on BxPC‐3 cells at months 6 and 12 (Figure S14b, Supporting Information). After 6 and 12 months of storage, PEG@Pac@PCN‐222 resulted in more toxicity at lower concentrations than the sample measured at time zero, while no visible variation was measured for concentrations above 0.1 µM. Considering that no toxic effect was shown from the empty material (Figure S14c, Supporting Information), the higher toxicity was probably related to some release of the drug in the solution over time. Overall, these results showed that the PEGylated formulation presents a high degree of stability in aqueous environments over a prolonged time. The observed stability of the MOF at just 4 °C for over a year offers advantages over other widely used drug delivery systems, such as liposomes and lipid nanoparticles, in terms of longevity and safety.^[^ ^66^ ^]^

## 7 In vivo Therapeutic Efficacy of PEG@Pac@PCN‐222

**Figure 5:**
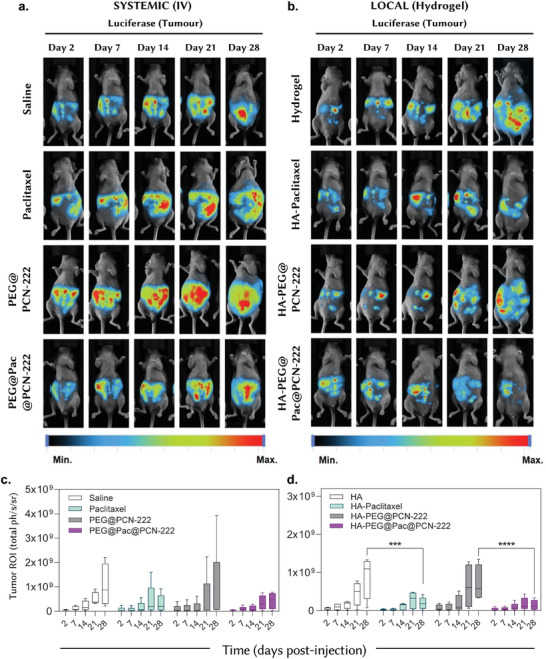
In vivo efficacy of free drug and PEG@Pac@PCN‐222 in a preclinical model of pancreatic cancer. a) Live imaging of athymic Balb/c female mice with pancreatic metastatic cancer upon intravenous administration. b) Live imaging of athymic Balb/c female mice with pancreaticmetastatic cancer upon local administration. c) Quantitative analysis of tumor growth by bioluminescence signal quantification of intravenously treated mice. d) Quantitative analysis of tumor growth by bioluminescence signal quantification of locally treated mice. Statistical analysis was performed using two‐way ANOVA with Tukey's multiple comparison test, ****P < 0.0001 and ***P < 0.001.

Following the positive in vitro response, we assessed the in vivo therapeutic efficacy of PEG@Pac@PCN‐222 against the free drug. In our preclinical pancreatic cancer model, we tested the vehicle and free drug via two routes: systemic intravenous (i.v.) and intraperitoneal (i.p.) local administration. Systemic intravenous administration offers the advantage of delivering the drug throughout the body, potentially targeting metastatic sites, whereas local administration focuses on maximizing drug concentration at the tumor site and reducing systemic exposure and associated side effects. Studying both methods allowed us to determine the most effective and safest delivery route for our MOF technology. To generate tumors, luciferase‐expressing BxPC‐3‐Luc human pancreatic cancer cells were injected intraperitoneally into athymic BALB/c female mice. This method mimics the dynamic progression of pancreatic tumors and allows for assessment using bioluminescence imaging. On day 21 post‐tumor induction, pancreatic cancer cells were mostly located in the hepatic, pancreatic, and peritoneal membrane regions. PEG@Pac@PCN‐222 and the free drug were then administered retro‐orbitally (i.e., intravenous systemic administration) or locally intraperitoneally, using a natural‐based hydrogel (4 wt.% HA). HA‐based hydrogels containing nanoparticles such as MOF are an efficient local delivery strategy, having prolonged contact with abdominal organs, reducing off‐target effects, and endowing a slightly significant tumor reduction on hydrogels containing the PEG@Pac@PCN‐222 formulation. Recently, it has been reported that the local delivery of NPs avoids the increased off‐target distribution of nanoconjugates compared to systemic administration while allowing a sustained release approach that can be more effective than intravenous injections.^[^ ^67^, ^68^, ^69^ ^]^ **Figure** **5** shows the in vivo therapeutic efficacy of the different formulations in a preclinical model of pancreatic cancer. We compared the systemic (i.v.) and local (hydrogel) administration routes of several mouse treatments. In the systemic (i.v.) administration panel (Figure 5a), the following treatments were evaluated: saline, paclitaxel, PEG@PCN‐222, and PEG@Pac@PCN‐222. Saline was the control group. Free paclitaxel showed some reduction in tumor size, but this was not significant over time. However, PEG@Pac@PCN‐222 showed the most significant reduction in tumor size, indicating the superior efficacy of the drug‐loaded MOF. Figure 5c presents a quantitative analysis of tumor growth by bioluminescence signal quantification, confirming the visual observations in Figure 5a. PEG@Pac@PCN‐222 showed the lowest tumor ROI, indicating the highest therapeutic efficacy among systemic treatments, reflecting a 74% reduction compared to the saline group and a 65% reduction compared to paclitaxel alone, although not statistically significant. In the local (hydrogel) administration panel (Figure 5b), the following treatments were compared: hydrogel, HA‐paclitaxel, HA‐PEG@PCN‐222, and HA‐PEG@Pac@PCN‐222. Hydrogel alone was used as the control group, with results similar to those of the systemic saline administration. HA‐paclitaxel displayed a significant reduction in tumor size, which was better than that of paclitaxel i.v. administration. HA‐PEG@Pac@PCN‐222 demonstrated the greatest reduction in tumor size among the locally administered formulations, indicating the highest efficacy. Figure 5d provides a quantitative analysis of tumor growth by bioluminescence signal quantification, confirming the visual results shown in Figure 5b. HA‐PEG@Pac@PCN‐222 showed the lowest tumor ROI, indicating the highest therapeutic efficacy among local treatments, representing a statistically significant 83% reduction compared to the hydrogel control and a 71% reduction compared to HA‐paclitaxel. To assess the impact of the treatments, and in particular the MOF system, we tracked the animals' weight over a 28‐day period, observing a positive trend with no significant weight loss, for both intravenous and intraperitoneal administration. This is a strong indication that the animals are feeding and drinking correctly, suggesting no side effects from the treatments (Figure S17, Supporting Information).

The efficacy comparison indicated that the PEG@Pac@PCN‐222 formulation showed the highest efficacy in reducing tumor size in both systemic and local administrations. This suggests that the encapsulation of paclitaxel within the PCN‐222 MOF and PEGylation significantly enhanced the therapeutic effect of the drug. While both administration routes showed significant therapeutic benefits, local administration (hydrogel) of HA‐PEG@Pac@PCN‐222 resulted in superior tumor reduction. This could be due to the sustained and concentrated drug release at the tumor site, thus minimizing systemic exposure and side effects. These data illustrate the superior therapeutic efficacy of PEG@Pac@PCN‐222, particularly when administered locally via hydrogel, highlighting its potential for effective pancreatic cancer treatment with reduced systemic toxicity.

**Figure 6:**
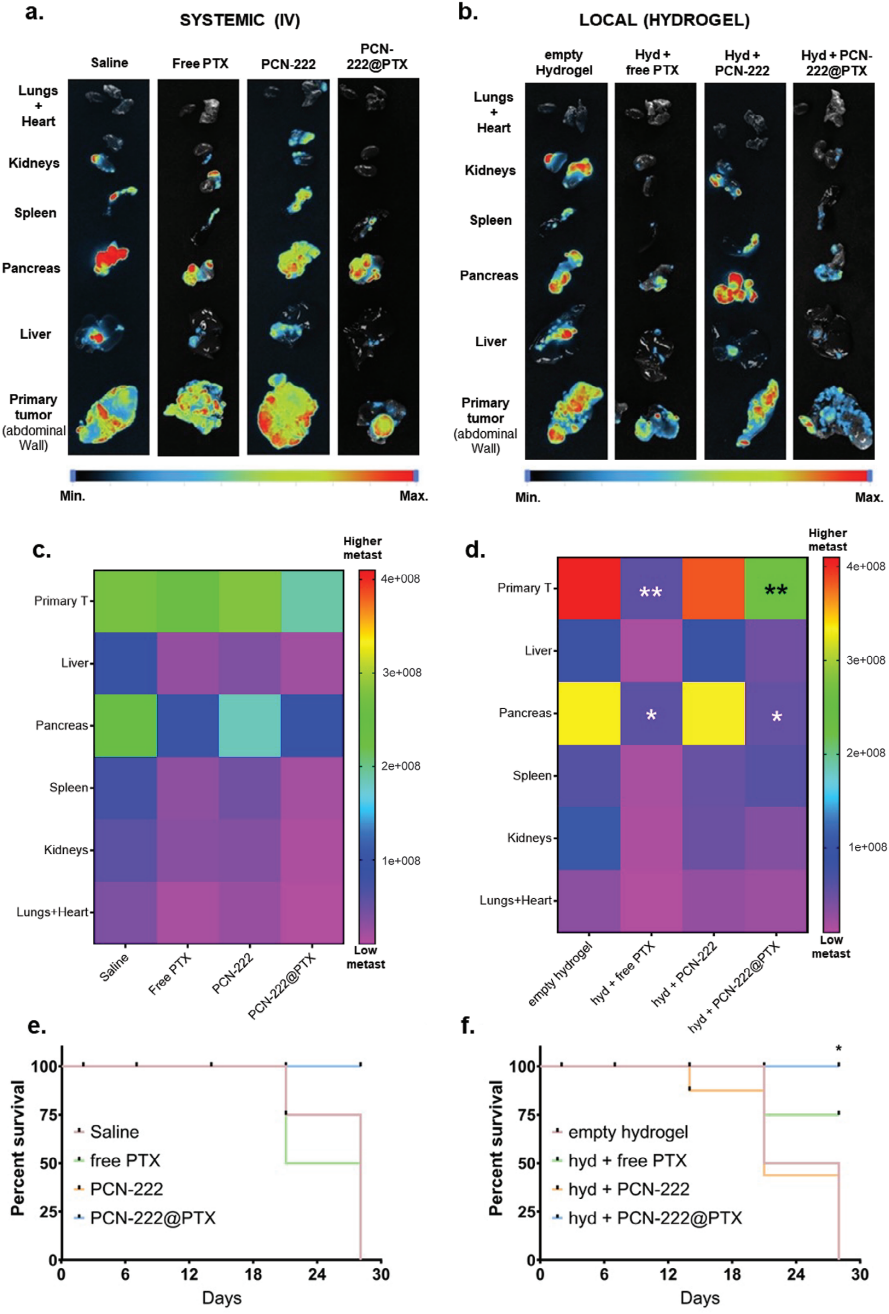
Tumor growth assessment of different mouse organs. Ex vivo bioluminescence imaging of organs extracted from a) an intravenously and b) locally administered preclinical model of pancreatic cancer to assess metastatic reduction. Heatmap representing the metastatic extension across different c. intravenously (i.v.) and d. locally (i.p.) treated mouse organs and the primary tumor (i.e., located in the abdominal wall). e,f) Survival study using Kaplan‐Meier plot of intravenous and intraperitoneal administration. Statistical analysis was performed using the log‐rank (Mantel‐Cox) test; *P < 0.05.

At the end of the in vivo study, the organs (lungs, heart, pancreas, spleen, and liver) and the abdominal wall (primary tumor) were extracted from the treated mice to evaluate the spread of pancreatic cancer metastasis. **Figure** **6**a,b shows the *ex vivo* bioluminescence imaging of organs extracted from the i.v. and i.p.‐administered PEG@Pac@PCN‐222, respectively. Both exhibited a reduced luminescence signal, which indicated a clear reduction in metastatic sites and primary tumor (i.e., abdominal wall) growth. In this in‐depth evaluation of PCN‐222 and its drug‐conjugate variant PEG@Pac@PCN‐222, the in vivo data illustrated the significant findings of a preclinical study on their impact on pancreatic cancer. Figure 6c,d shows heat maps representing the metastatic spread across different organs in mice treated with either i.v. or i.p. administration of PEG@Pac@PCN‐222. In addition, we also quantified the relative areas of pancreas and primary tumors using the bioluminescence signal data (Figure S18, Supporting Information). The heat maps provide a visual comparison of the extent and intensity of metastatic dissemination in different organs following treatment. The color gradients, ranging from blue (minimal metastasis) to red (maximum metastasis), allow for a quick assessment of treatment efficacy. In the systemic (i.v.) administration (Figure 6c; Figure S18, Supporting Information), the metastatic spread is more pronounced, particularly in the lungs, liver, and pancreas. Conversely, the local (i.p.) administration (Figure 6d; Figure S18, Supporting Information) demonstrates a more concentrated and localized effect, significantly reducing the tumor in the pancreas and liver. This indicates that the hydrogel‐based local delivery system enhances the therapeutic impact at the tumor site while minimizing off‐target effects and systemic toxicity, which is consistent with the reduced luminescence signals observed in the corresponding organs. We also assessed the survival rate of the mice following intravenous and intraperitoneal administration, as shown in Figure 6e,f, respectively. In both administration routes, the treated mice exhibited a higher survival rate with the hyd‐PCN‐222@PTX/hyd and PCN‐222@PTX formulations, showing lower toxicity compared to the free PTX/hyd + free PTX treatments. We also performed histopathological analysis of the collected organs (lung, liver, kidney, heart, and spleen) to evaluate the biocompatibility of PCN‐222, following intravenous administration (Figure S19, Supporting Information). The inflammatory profile was assessed by hematoxylin‐eosin staining. Notably, after 14 days, PCN‐222 displayed a normal inflammatory profile in the lung, kidney, heart, and spleen tissues. In the liver, minimal activation of Kupffer cells with ceroid pigment was observed in two animals, while the inflammatory profile remained within normal limits in one animal.

Local administration facilitated by the hydrogel demonstrated a superior ability to maintain a more prolonged and concentrated interaction with target regions, suggesting an enhanced therapeutic impact with reduced systemic dispersion and toxicity. These results are consistent with recent reports advocating local delivery to circumvent the undesirable systemic distribution often associated with intravenous methods.^[^ ^70^ ^]^ These findings highlight the promising potential of PCN‐222‐based frameworks in cancer therapy and emphasize the strategic advantages of localized drug delivery systems. Evidence supports advancement toward such localized treatment methods, which could improve the clinical management of pancreatic cancer and potentially other metastatic malignancies.

## 8 Conclusion

This study demonstrates the rational selection and development of drug‐loaded PCN‐222 and its translation into preclinical applications. High‐throughput screening of 143 MOFs identified PCN‐222 as one of the best candidates for the loading of gemcitabine, paclitaxel, and SN‐38 owing to its high porosity and biocompatibility. Taking into account practical wet‐lab aspects such as ease of synthesis and adjustable particle size inclined the balance toward this material. Following the successful loading and characterization of the three drug‐loaded MOF, in vitro assays confirmed the dose‐dependent toxicity of drug‐loaded PCN‐222 in three different pancreatic cancer cell lines, with reduced toxicity compared with the free drug. This reduction in toxicity was attributable to the PCN‐222 slow‐release properties, as shown in the in vitro assays. Compelling in vivo data confirmed the distinct advantages of PEGylated PCN‐222 nanoparticles, particularly when conjugated with paclitaxel (PEG@Pac@PCN‐222), for the targeted treatment of pancreatic cancer. These findings demonstrate the high efficacy of both systemic and local administration routes in inhibiting tumor growth. Local administration of the HA‐based hydrogel resulted in a marked reduction in the metastatic spread and primary tumor development, attributed to its sustained release and minimized systemic dispersal, as evidenced by the reduced bioluminescent signal in the extracted organs. Such strategies exemplify the potential of localized delivery systems to improve the therapeutic index of nanoparticle‐mediated drug delivery by focusing on the therapeutic action at the disease site while limiting systemic toxicity. Most importantly, the long‐term stability of the drug‐loaded MOF, which maintains its integrity in aqueous solution for over a year, is a promising indicator of the shelf life and durability of this formulation. Our study showed that MOF‐based formulations, particularly PCN‐222, could resolve the solubility limitations of hydrophobic drugs such as gemcitabine, paclitaxel, and SN‐38. This paves the way for new drug formulations that can significantly broaden the therapeutic window and improve the quality of treatment for patients undergoing chemotherapy. The prospect of employing such nanocarrier systems has implications for cancer nanomedicine. Integrating advanced materials such as MOFs with the precision of localized delivery promises to address the heterogeneity and associated therapeutic challenges in pancreatic cancer. It is imperative to continue refining these nanoparticle formulations and delivery methods to ensure their translation from preclinical models to clinical settings. Further research on the structure of MOFs, drug release mechanisms, long‐term stability, biodegradability, potential immune responses, and direct comparative studies with other drug delivery systems are crucial to fully understanding their advantages and limitations. This will help tailor MOF formulations to meet therapeutic needs effectively by balancing their stability, biocompatibility, and efficacy. The convergence of material science, nanotechnology, and oncology has transformed the current cancer treatment paradigm, particularly for hard‐to‐treat cancers such as pancreatic cancer, where every incremental advance can translate to significant improvements in patient survival and quality of life.

## Conflict of Interest

D.F.‐J. is co‐founder of Vector Bioscience Cambridge, working on the commercialization of MOFs in healthcare applications. J.C. is a co‐founder and shareholder of TargTex S.A. Targeted therapeutics for Glioblastoma Multiforme. J.C. is a member of the Global Burden Disease (GBD) consortium from the Institute for Health Metrics and Evaluation (IHME), University of Washington (US). The remaining authors declare no competing interests.

## Supporting information



Supporting Information

Supplemental Table 1

## Data Availability

The data that support the findings of this study are available in the supplementary material of this article.
