# Supplementary material for: Rational Design of Metal–Organic Frameworks for Pancreatic Cancer Therapy: from Machine Learning Screening to In Vivo Efficacy
Source: Adv Mater. 2025 Feb 2;37(52):2412757. doi: 10.1002/adma.202412757 (PMC12747470; doi:10.1002/adma.202412757)
Supplement: Supplementary file 1 — Supporting Information [file ADMA-37-2412757-s001.pdf]

ADVANCED MATERIALS

Supporting Information

for *Adv. Mater.*, DOI 10.1002/adma.202412757

Rational Design of Metal–Organic Frameworks for Pancreatic Cancer Therapy: from Machine Learning Screening to In Vivo Efficacy

Francesca Melle, Dhruv Menon, João Conniot, Jon Ostolaza-Paraiso, Sergio Mercado, Jhenifer Oliveira, Xu Chen, Bárbara B. Mendes, João Conde and David Fairen-Jimenez**

Supporting Information for:

Rational Design of Metal-Organic Frameworks for Pancreatic Cancer Therapy: From Machine Learning Screening to *In Vivo* Efficacy

Francesca Melle[a], Dhruv Menon[a], João Conniot[b,c], Jon Ostolaza-Paraiso[a], Xu Chen[a], Sergio Mercado[a], Jhenifer Oliveira[b,c], Bárbara B. Mendes[b,c], João Conde[b,c,*] and David Fairen-Jimenez[a,*]

[a]Adsorption & Advanced Materials Laboratory (AAML), Department of Chemical Engineering & Biotechnology, University of Cambridge, Philippa Fawcett Drive, Cambridge CB3 0AS, UK

[b]NOVA Medical School|Faculdade de Ciências Médicas, NMS|FCM, Universidade NOVA de Lisboa, Lisbon, Portugal

[c]ToxOmics, NOVA Medical School|Faculdade de Ciências Médicas, NMS|FCM, Universidade NOVA de Lisboa, Lisbon, Portugal

*Corresponding authors: joao.conde@nms.unl.pt; df334@cam.ac.uk

Table of Contents

S1. Computational details **S2**
S2. General synthetic procedures and protocols **S5**
S3. Material characterization **S10**
S4. *In vitro* methods **S14**
S5. Long-term stability **S20**
S6. BET area calculation using BETSI **S21**
S7. *In vivo* studies **S23**
S8. References **S24**

S1. Computational details.

Machine Learning (ML) screening: The ML methods used here were recently developed and reported by us. Here, we briefly discuss the methodology that was followed previously – for in-depth discussions however, we would request the reader to refer to the source publication.[1] For assessing the biocompatibility of a MOF, we evaluated the toxicity of its building blocks. For the metal center, we curated a comprehensive database of the reported median lethal dose (LD_{50}) of the corresponding chloride salt, when administered to rats orally. For the organic linker, we developed Random Forest (RF) based classification algorithms based on the United Nations 'Globally Harmonized System of Classification and Labelling of Chemicals' (UN GHS)[2] (discussed in the Ref.[1]). The UN GHS classifies chemicals into five categories based on the median lethal dosage (LD_{50}): **Category 1** if $LD_{50} < 5$ mg g^{-1}; **Category 2** if 5 mg $g^{-1} < LD_{50} < 50$ mg g^{-1}; **Category 3** if 50 mg $g^{-1} < LD_{50} < 300$ mg g^{-1}; **Category 4** if 300 mg $g^{-1} < LD_{50} < 2000$ mg g^{-1}; and **Category 5** if 2000 mg $g^{-1} < LD_{50}$. Chemicals belonging to **Categories 1** and **2** are generally 'fatal', **Categories 3** and **4** are generally 'toxic', while **Category 5** are generally safe. Here, for a more robust and accurate classification, we defined three concentration thresholds for both the metallic centre and the organic linker corresponding to 'fatal' (**Categories 1** and **2**), 'toxic' (**Categories 3** and **4**) and 'safe' (**Category 5**). These algorithms were developed for the intraperitoneal (i.p) and oral routes of administration. Based on these models, we screened the Cambridge Structural Database (CSD)[3] which contains *ca.* 86,000 non-disordered MOF structures. The screening was first done on the basis of the metal center, followed by the organic linker. For extracting the organic linker from the crystallographic information file (CIF) of the MOF, we used the *moffragmentor* library developed by Jablonka et al.[4] In some cases, despite having a 'safe' center, MOFs also contain toxic metals such as Cd or Ni. Thus, during the fragmentation procedure, these MOFs were eliminated from consideration. These extracted organic linkers were then assessed using the classification models, yielding 'fatal', 'toxic' and 'safe' linkers. MOFs that contained a 'safe' metal center and organic linker were thus deemed highly biocompatible.

With the exception of Zr-centered MOFs, most highly biocompatible MOFs are non-porous. Since non-porous MOFs will not uptake significant quantities of guest molecules (i.e. drugs), these MOFs were eliminated from consideration, leaving behind a modest subset of porous, yet biocompatible MOFs. To this subset, due to their widespread usage in drug delivery, we included porous Fe-centered MOFs with 'safe' linkers, again, deduced from the classification algorithm, along with commonly used Zn-MOFs such as ZIF-8 and ZIF-90. This brought the candidate structures to 143 MOFs, featuring prominent MOFs such as ZIF-8, MIL-88(Fe), MOF-808, UiO-66, PCN-128, PCN-222, PCN-224 and PCN-777, among others. In **Table S1** we provide the list of MOFs investigated during the subsequent molecular simulations along with their textural properties as calculated using Zeo++ with a He probe.[5]

Grand canonical Monte Carlo (GCMC) simulations: The GCMC simulations were performed using RASPA.[6] Each simulation was performed at a fugacity of 1 bar to ensure that the systems were saturated to ascertain the maximum possible drug loading. Gemcitabine, Paclitaxel, and SN-38 were modeled using the OPLS-AA (optimized potentials for liquid simulations – all atom) force field as it is optimized for organic liquids.[7] Due to the inability of RASPA to accommodate flexible rings, each drug was modeled as a partially flexible molecule, keeping the backbone completely rigid. **Figure S1** shows a schematic illustration of the modeling procedure for gemcitabine as a representative example. Bonds and angles not part of the rigid backbone were allowed to stretch and bend, respectively, according to the functional form shown below,

$$\text{Bond Stretching: } E_{bond} = \sum_{bonds} K_r \left(r - r_{eq}\right)^2$$

$$\text{Angle Bending: } E_{angle} = \sum_{angles} K_\theta \left(\theta - \theta_{eq}\right)^2$$

While OPLS-AA also accounts for torsional effects, it was found that the introduction of torsional parameters was significantly increasing the computational cost, while not having a significant effect on the final loading capacities, and were thus, not considered during the screening. The atomic coordinates for each drug molecule were extracted from DrugBank,[8] following which the molecules were geometry optimized using the semi-empirical VAMP module in Materials Studio.[9] **Figure S2** shows the labels assigned to each atom in the drug molecules, while input scripts that contain the relevant force-field parameters have been appended. These force-field parameters were extracted using the LigParGen server wherein charges were assigned using the 1.14*CM1A[1] scheme.[10–12]

In the GCMC simulations we performed, four moves were considered: insertion, deletion, translation, and rotation of the drug molecules inside the framework. The chances of each move occurring were assigned an equal probability. Each simulation was run over 10,000 cycles to first equilibrate the system, followed by 100,000 production cycles to average the properties. A single cycle is defined as the maximum of 20 steps or the number of molecules in the system – to allow for a Monte Carlo move on all molecules during each cycle. Since, in principle, GCMC samples the free-energy landscape over a large number of cycles, it provides a good estimation of the drug loading in the MOF.

For the high-throughput screening, the CIF of the MOFs under consideration were extracted from the CSD. Prior to running the drug loading calculations, we calculated the Helium void fraction using RASPA. For the partial charges on the framework atoms, we used the EQeq method.[13] During the simulations, the framework atoms were considered rigid (that is, they were fixed at their crystallographic positions). Intermolecular interactions were modeled using the Lennard-Jones (LJ) potential, with a cutoff of 12.8 Å. Cross-interaction terms were accounted for using the Lorentz-Berthelot mixing rules. To avoid any finite-size effects, the simulation supercell was considered with dimensions of at least twice the cutoff of the long-range interactions. The electrostatic interactions were modeled using the coulombic potential as computed using the Ewald summation method with

a precision of 1e-06. The framework atoms were modeled with LJ parameters using a combination of the Universal force field (UFF) and DREIDING force field parameters[14,15] – again appended separately.

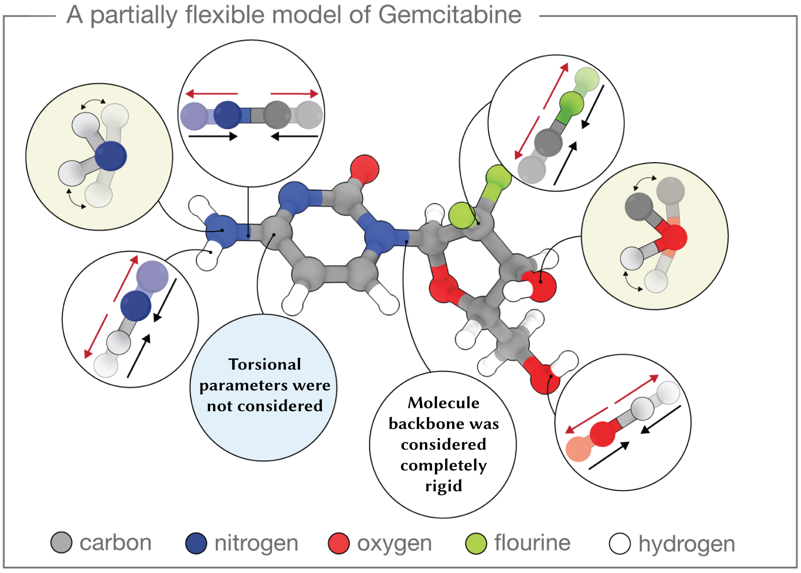

Figure S1. A schematic illustration of the modeling procedure for the drug molecules. For all three drugs, a partially flexible model was developed wherein the molecule backbone was considered completely rigid, while bonds and angles not part of the backbone were allowed to stretch and bend, respectively, according to the functional form of the force-field used. Additionally, torsional parameters were not included in the modeling.

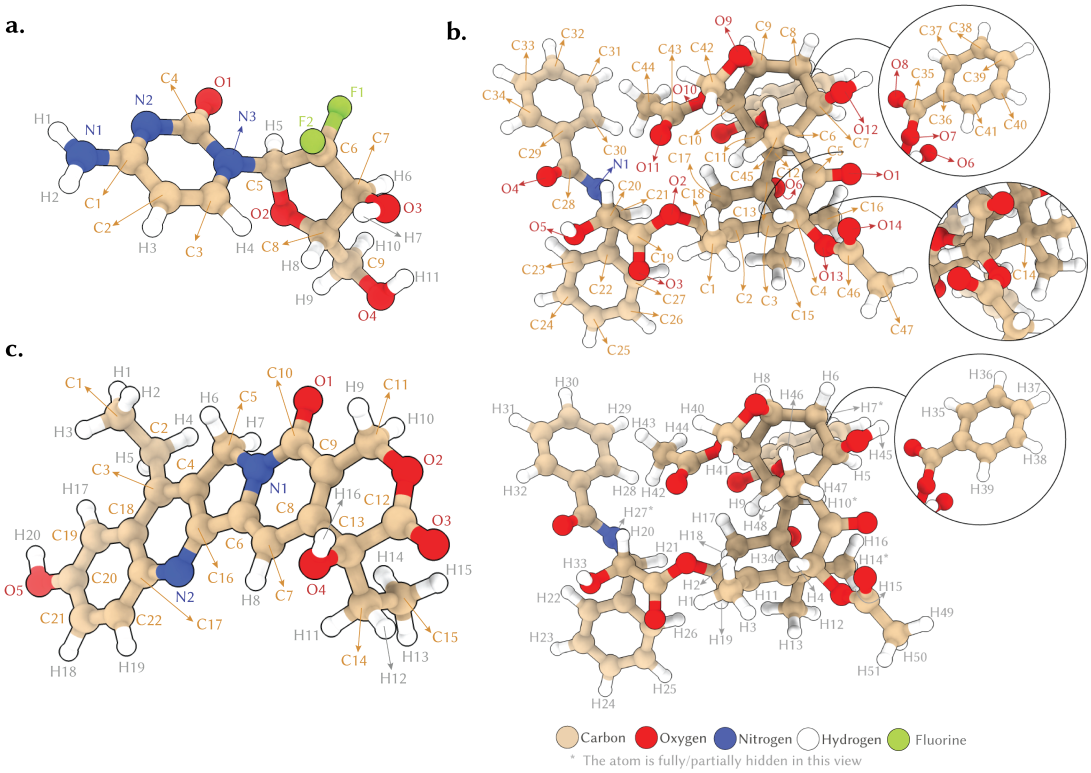

Figure S2. Atom numbers assigned to **a.** Gemcitabine **b.** Paclitaxel and **c.** SN38. The corresponding force-field parameters and partial charges have been described in the input scripts provided.

Table S1. Drug-loading capacities of the MOFs considered in the high-throughput screening along with their textural properties. The textural properties were calculated using Zeo++ with a He probe.

Note: Due to the large size of the table, we have appended it as a .csv file. Additionally, sample input scripts which contain the force-field parameters, molecule definitions and partial charges have been appended.

S2. General synthetic procedures and protocols

All chemicals, unless otherwise stated, were purchased from commercial suppliers, and used as received. Milli-Q water (18.2 MΩ.cm resistivity at 25 °C) was used throughout the experiment. Dialysis tubing (Molecular Weight Cut-off; MWCO 3,500 and 12,000 - 14,000 Daltons) was obtained from Medicell Membranes Ltd. The synthesis of nanosized PCN-222 and mPEG5K-phosphate (mPEG-PO_3) were adapted from the reported procedures. [16,17]

Drug loading and PEGylation

The drug solutions (5 mg/ml) were prepared by dissolving gemcitabine, paclitaxel, and SN-38 in 10 ml of water, ethanol, and DMSO, respectively, using a 20 ml glass vial. The drug concentrations were measured using UV-Vis and calculated using previously made calibration curves (**Figure S5**). 20 mg of PCN-222 (10 mg/ml) were resuspended in 2 ml of water, ethanol, and DMSO for the respective loadings of gemcitabine, paclitaxel, and SN-38. The PCN-222 was mixed with the respective drug solution dropwise and stirred at RT for 72 h. Following drug encapsulation, the drug@PCN-222 was collected and washed by centrifugation (16,000 rpm for 1 h) several times to remove the unloaded drugs completely. For the Gem@PCN-222, the three washes were all carried in water, Pac@PCN-222 was resuspended two times in ethanol and one time in water, and SN38@PCN-222 was resuspended two times in DMSO, one time in methanol and finally in water, to make sure that the unloaded drug and the solvent were completely removed. All the supernatants were collected and measured using UV-Vis, while the three pellets were resuspended in 10 ml of water-PEG-solution (20 mg/ml) and stirred at RT for 48 h. Following PEGylation, the samples were collected, inserted into a dialysis bag, and exchanged against water four times over two days to remove the unreacted PEG. After dialysis, all the samples were collected and characterized by DLS, z-potential, SEM, PXRD, FT-IR, and TGA. The concentration of the final formulation, PEG@drug@PCN-222, was measured, and the drug loading (wt.%) was calculated using the formula:

$$\text{drug loading wt.}\,(\%) = \frac{\text{encapsulated drug (mg)}}{\text{PEG@drug@PCN} - 222\ \text{(mg)}} \cdot 100$$

where $encapsulated\ drug$ = $drug\ [stock\ solution](mg) - drug\ [supernatant](mg)$

Powder X-ray diffraction (PXRD): PXRD measurements were carried out at 298 K using a PANalytical X'Pert PRO diffractometer (λ (CuKα)=1.4505 Å) on a mounted bracket sample stage. Data were collected over the range 2–30 2θ. The calculated patterns were produced using the Mercury program and single crystal diffraction data.

Dynamic light scattering (DLS) and zeta-potential: DLS Measurements were performed using a Zetasizer Nano ZS (Malvern Instrument Ltd., UK) equipped with a He−Ne laser operating at 633 nm at 25°C. Z-potential was measured using folded capillary Zeta cell DTS1070. The Smoluchowski equation was used to calculate the z-potential. Measurements were performed three times with over 10 subruns for each sample. Error bars represent the standard deviation of three measurements. All the samples were recorded using an aqueous solution with a 0.2 mg/ml concentration.

Scanning electron microscopy (SEM): SEM was used to analyze MOFs' structure and surface topology before and after surface functionalization with PEG. Samples were coated with Pt or Au for 40 seconds and imaged using a FEI Nova Nano SEM 450. Particle size distribution was analyzed manually with at least 200 measurements for each sample, using ImageJ software.

Fourier-transform Infrared Spectroscopy (FT-IR): FT-IR was carried out using a Bruker Tensor 27 FTIR with attenuated total reflectance (ATR) method.

Thermogravimetric analysis (TGA): TGA measurements were carried out for thermal stability using a TA Instruments Discovery TGA5500. Measurements were collected from room temperature to 800 °C with a heating rate of 5 °C / min under air with a 25 mL/min flow rate.

Gas adsorption experiments: Characterisation through N_2 adsorption was measured on either a TriStar II or 3Flex (Micromeritics) machine. For samples run on TriStar II, activation occurred in a sorption tube at 120°C for 12 h under vacuum on a SmartVacPrep (Micromeritics). For the samples run on 3Flex, degassing occurred at 120°C for 10 h. The nitrogen isotherm was measured from P/P0=0 to 0.99 at 77 K (held by liquid nitrogen bath).

UV-Vis Spectroscopy: UV-vis and fluorescence spectra were recorded using a Tecan Spark® Multimode Microplate Reader.

Cell culture: Human pancreatic cancer cell lines BxPC-3, MIA-PaCa-2, and PANC-1 were purchased from the American Type Culture Collection (ATCC). MIA-PaCa-2 and PANC-1, were grown in DMEM (Sigma, UK) supplemented with 10% (v/v) FBS, 100 units/ml penicillin, and 100 µg/ml streptomycin. BxPC-3 cells were cultured using RPMI-1640 supplemented with 10% (v/v) FBS, 100 units/ml penicillin, and 100 µg/ml streptomycin. All cell lines were cultured in a humidified environment at 37°C with 5% CO_2. All cell lines were routinely tested to confirm the absence of mycoplasma and verified by STR profile. *In vitro* experiments were conducted with 60% to 80% confluent cultures at passage numbers between 5 and 15.

Live-cell cytotoxicity studies: All drug-loaded PCN-222 formulations and bare PCN-222 were tested for cytotoxicity studies in BxPC-3, MIA-PaCa-2, and PANC-1 cell lines. Cells were seeded into 96-well plates at a concentration of 30,000 cells/ml (MIA-PaCa-2), 35,000 cells/ml (BxPC-3 and PANC-1), in 100 µl of complete growth medium and incubated at 37°C, 5% CO_2 for 24 h. After overnight incubation, the cells were treated with different concentrations of PEG@PCN-222 (1-500 µg/ml), PEG@Gem@PCN-222, PEG@Pac@PCN222, PEG@SN38@PCN-222 (0.001-10 µM) and the respective DMSO dissolved drug controls gemcitabine, paclitaxel and SN-38, all dissolved in complete cell media. The plates were then inserted into the Incucyte S3 Live-Cell Analysis System (Sartorius) for real-time imaging. Treated plates were imaged every 3 h for 72 h under cell culture conditions with 10x objective using the brightfield channel. Mean cell confluence was calculated using the images taken from three random fields of view per well using the Incucyte S3 v2022A software. All Incucyte experiments were performed in triplicates in three independent experiments. Relative confluence values were obtained by normalizing each value to the time zero value in each sample and normalized it to the untreated control sample.

$$\text{Confluence (\%)} = 100 \cdot \frac{\text{confluence [treated cells 72 h]} - \text{confluence [treated cells 0 h]}}{\text{confluence [untreated cells 72 h]} - \text{confluence [untreated cells 0 h]}}$$

MTS cytotoxicity studies: The effect of PEG@PCN-222, PEG@Gem@PCN-222, PEG@Pac@PCN-222, PEG@SN38@PCN-222, and free drugs (gemcitabine, paclitaxel and SN-38) on the viability of BxPC-3, MIA-PaCa-2, and PANC-1 cell lines was evaluated using MTS assay (Promega, USA). Cells were seeded into 96-well plates at 3,000 cells/well (MIA-PaCa-2), 3,500 cells/well (BxPC-3 and PANC-1) in 100 µl of complete growth medium and incubated at 37°C, 5% CO_2 for 24 h. Subsequently, cells were treated with varying concentrations of free drugs (gemcitabine, paclitaxel, and SN-38) dissolved in complete growth media containing 1% of DMSO or loaded MOFs (PEG@PCN222 PEG@Gem@PCN222, PEG@Pac@PCN222, PEG@SN38@PCN222) dissolved in complete growth media containing 1% of water. After 72 h of incubation, samples were washed three times with 1xPBS, mixed with 100 µl of complete growth media containing MTS solution, and incubated in the dark at 37°C, 5% CO2 for 1-4 h according to the manufacturer's instruction. The absorbance of each well was measured at 490 nm using a Spark plate reader (TECAN, CH). Control measurements included negative control of cells with DMEM, cells with DMEM containing 1% water or 1% DMSO, and cell-free cultured media (blank). All experiments were conducted in biological triplicates. The percentage of cell viability was calculated according to the following:

$$\textit{Cell viability}\ (\%) = 100 \cdot \frac{\text{absorbance [treated cells 72 h]} - \text{absorbance [blank]}}{\text{asorbance [untreated cells 72 h]} - \text{absorbance [blank]}}$$

MTS cytotoxicity studies (short-term): PEG@PCN222, PEG@Gem@PCN222, PEG@Pac@PCN222, PEG@SN38@PCN222, and free drugs (gemcitabine, paclitaxel and SN-38) were dosed for a short-incubation time on BxPC-3, MIA-PaCa-2, and PANC-1 cell lines and tested using MTS assay (Promega, USA). Briefly, Cells were seeded into 96-well plates at 10,000 cells/well

in 100 µl of complete growth medium and incubated at 37°C, 5% CO_2 for 24 h. Subsequently, cells were treated with varying concentrations of free drug (Gemcitabine, Paclitaxel, and SN-38) dissolved in complete growth media containing 1% of DMSO or loaded MOFs (PEG@PCN222 PEG@Gem@PCN222, PEG@Pac@PCN222, PEG@SN38@PCN222) dissolved in complete growth media containing 1% of water. Following 6 h of incubations, samples were washed two times with full media and incubated at normal growing conditions for seven days, changing the media every three days. During this time, cells were co-incubated with CytotoxRed dye (Incucyte, Sartorius) and imaged every hour using the Incucyte system. At the end of the seven days, cells were assessed via MTS assay as described before.

Flow cytometry: Cells were seeded into a 24-well seeded at 50,000 cells/well concentration and incubated at 37°C 5% CO_2 for 48 h. After incubation, cells were dosed with different concentrations of PEG@PCN-222 for different time points. At the end of the incubation, cells were washed twice with PBS, harvested by adding 100 µl of trypsin and incubated at 37°C 5% CO_2. Cells were then recovered by centrifugation (5 minutes at 12,000 rpm) and resuspended in 0.5 ml of PBS+4% FBS solution and measured using flow cytometry. Flow cytometry data were acquired with an LSR FortessaTM (BD Biosciences) cell analyser. Cells were gated by forward and side scatter to isolate single cells from debris and cell doublets. Unless specified, at least 10,000 events were acquired for the cell population of interest. FlowJo (Tree Star) and Prism (GraphPad, USA) software were used to analyse the data.

Confocal microscopy: Cells were seeded into an 8-well Nunc™ Lab-Tek™ II Chamber Slide™ System Measurements at a concentration of 100,000 cells/ml and incubated at 37°C for 24 h, then treated with water (control) or PEG@PCN-222 at a concentration of 100 µg/ml for 24 h at 37°C. After three washes with 1xPBS, the cells were stained with CellMask™ Green (Thermo Fischer) Plasma membrane stain and Hoechst 33342 (Thermo Fisher) according to the manufacturer's instructions. Cells were then washed gently three times with 1xPBS and imaged using a confocal microscope (Axio Observer Z1 LSM 800, Zeiss) equipped with 405, 488, and 561 nm lasers. The 405 and 514 nm lasers were used to excite MOFs and the cell mask stain, respectively, with data collected whilst using an oil immersion 63x lens. Zen software (Zeiss) was used for acquisition and image processing.

***In vivo* studies.** All animal experiments were approved by the Ethical Committee and the Animal Welfare and Ethics Body of Nova Medical School (21_01_ORBEA.4) and followed the Animal Research guidelines of Nova Medical School and the Directorate-General for Food and Veterinary Medicine. Athymic BALB/c female nude mice (6 weeks old, average weight = 20 g) were purchased from Charles River Laboratories (France) and acclimatized for 1 week before the experiments. Animals were kept under aseptic conditions with light/dark cycles of 12h. Water and standard pellet food were provided ad libitum. For pancreatic tumor induction, luciferase-expressing BxPC-3-Luc human pancreatic cancer cells ($2.4x10^6$/mouse) were injected intraperitoneally and tumor growth

was monitored using bioluminescence imaging. Briefly, mice were injected intraperitoneally with D-Luciferin (ABP Biosciences, 15 mg/ml in DPBS, 2000 µl/mouse). After 1 min, a Newton FT500 imaging system (Vilber) was used for bioluminescence imaging. At day 21 post tumor induction, mice were distributed in 8 groups (n=5): 1) Saline, 2) free paclitaxel, 3) PCN-222, 4) PEG@Pac@PCN-222, 5) Hydrogel, 6) Hydrogel with free paclitaxel, 7) Hydrogel with PCN-222, and 8) Hydrogel with PEG@Pac@PCN-222. Groups 1, 2, 3, and 4 received 100 µl/mouse (50ul in each side) of each formulation via retro-orbital intravenous administration. Groups 5, 6, 7, 8 received 500µl/mouse of the hyaluronic acid hydrogel containing the respective treatment by intraperitoneal injection with a 23G needle. Bioluminescence measurements were performed on days 2, 7, 14, 21, and 28 post treatment. The bioluminescence signal was also used to quantify the area of the pancreas and primary tumors using Kuant software. At the endpoint (day 28), mice were euthanized by cervical dislocation. The abdominal wall (i.e., primary tumor) and organs (liver, spleen, pancreas, heart, lungs, and kidneys) were harvested, imaged for bioluminescence, and then fixed in 10% formalin for further histological studies.

Biocompatibility studies. SKH1 Hairless Mouse (6 weeks old, average weight = 20 g) were purchased from Charles River Laboratories (France) and acclimatised for 1 week before the experiments. Animals were kept under aseptic conditions with light/dark cycles of 12h. Water and standard pellet food were provided *ad libitum*. The animal procedures were performed under isoflurane anaesthesia (IsoFlo 100% p/p). Mice were randomized in two groups (10 animals, 5 per group): 1) control (PBS) and 2) PCN-222. Mice received 7,5 mg/mL by retro-orbital injection. At the endpoint (day 14), mice were euthanised by cervical dislocation. Liver, spleen, pancreas, heart, and lungs were harvested and fixed in 4% formalin for histological analysis. The presence of epithelial damage in mouse organs was accessed by hematoxylin-eosin staining. Briefly, formalin-fixed paraffin embedded (FFPE) samples were sectioned at 3 µm thickness, mounted on positively charged glass slides and dried overnight at 65°C. After deparaffinization, samples were rehydrated and stained with haematoxylin and eosin (H&E). All animal experiments were approved by the Ethical Committee and the Animal Welfare and Ethics Body of Nova Medical School (21_01_ORBEA.4) and followed the Animal Research guidelines of Nova Medical School and of the Directorate-General for Food and Veterinary Medicine.

Statistical Analysis. All experiments were performed at least three times, and the data are presented as the mean ± SD. All statistical analyses were calculated using GraphPad Prism (9.0, GraphPad Software, San Diego, CA, USA). The statistical comparisons between multiple groups were performed using one-way analysis of variance (ANOVA) or two-way ANOVA. For all tests, $p < 0.05$ was determined as statistically significant.

S3. Material characterization

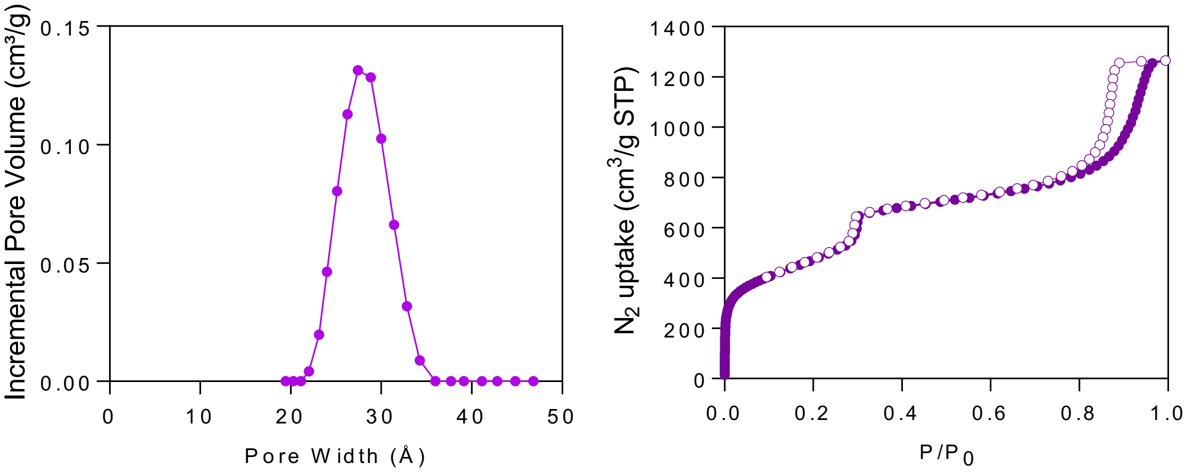

Figure S3. Pore size distribution analysis of PCN-222 (left) and Experimental N_2 isotherms at 77 K (right).

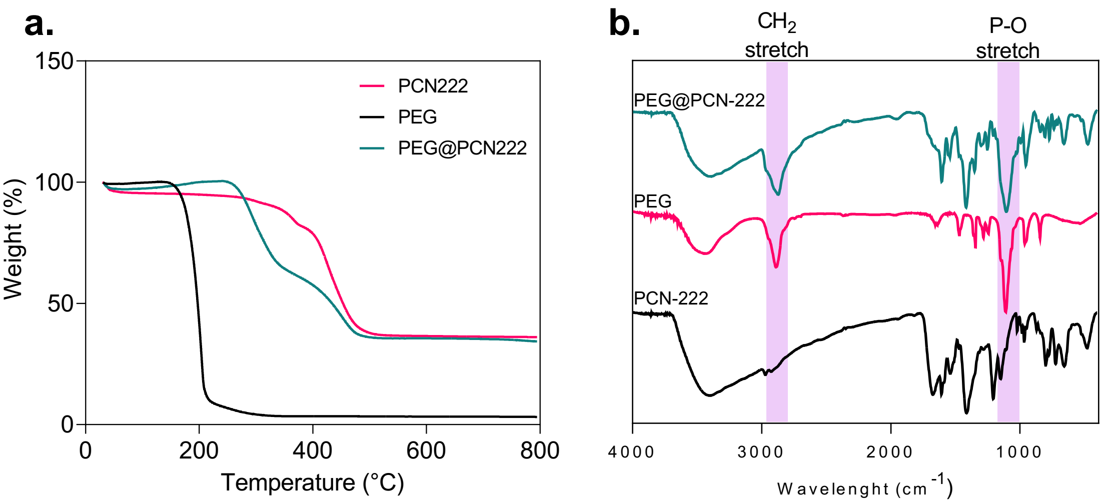

Figure S4. TGA and FT-IR analysis of PEG@PCN222. a) TGA analysis: the weight-loss curve of PEG@PCN-222 was compared to the ones of PCN-222 and PEG. b) FT-IR spectra were measured for PEG@PCN-222, PCN-222, and PEG. Colored rectangles highlight the characteristic peaks of PEG (2,866 and 1,088 cm-1 corresponding to the stretching vibrations of the C-H and P-O.

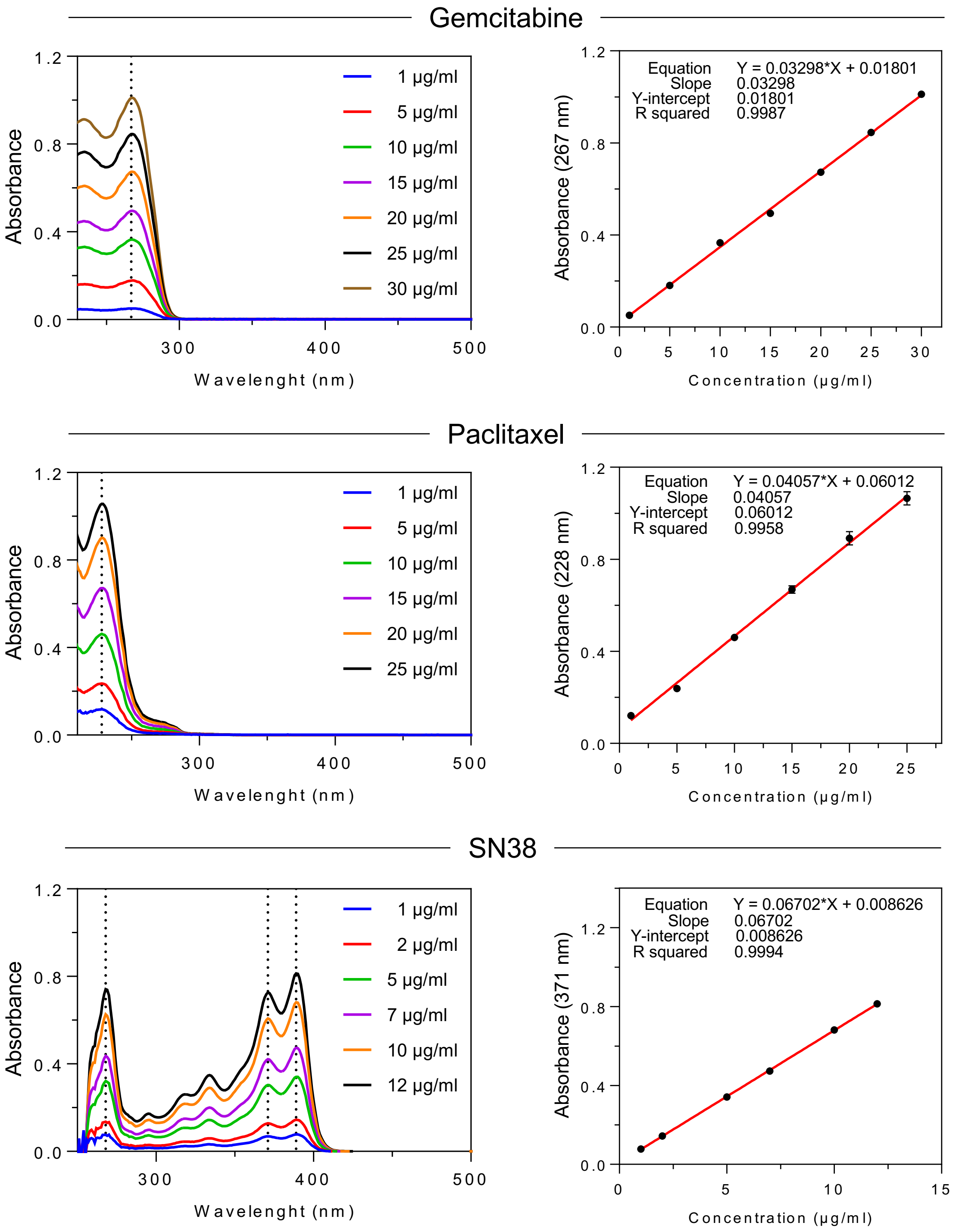

Figure S5. Absorbance spectra and calibration curves of gemcitabine, paclitaxel, and SN-38 measured with UV-Vis. Gemcitabine, paclitaxel, and SN-38 were dissolved in water, ethanol, and DMSO respectively. Calibration curves were calculated in triplicates; error bars represent SD.

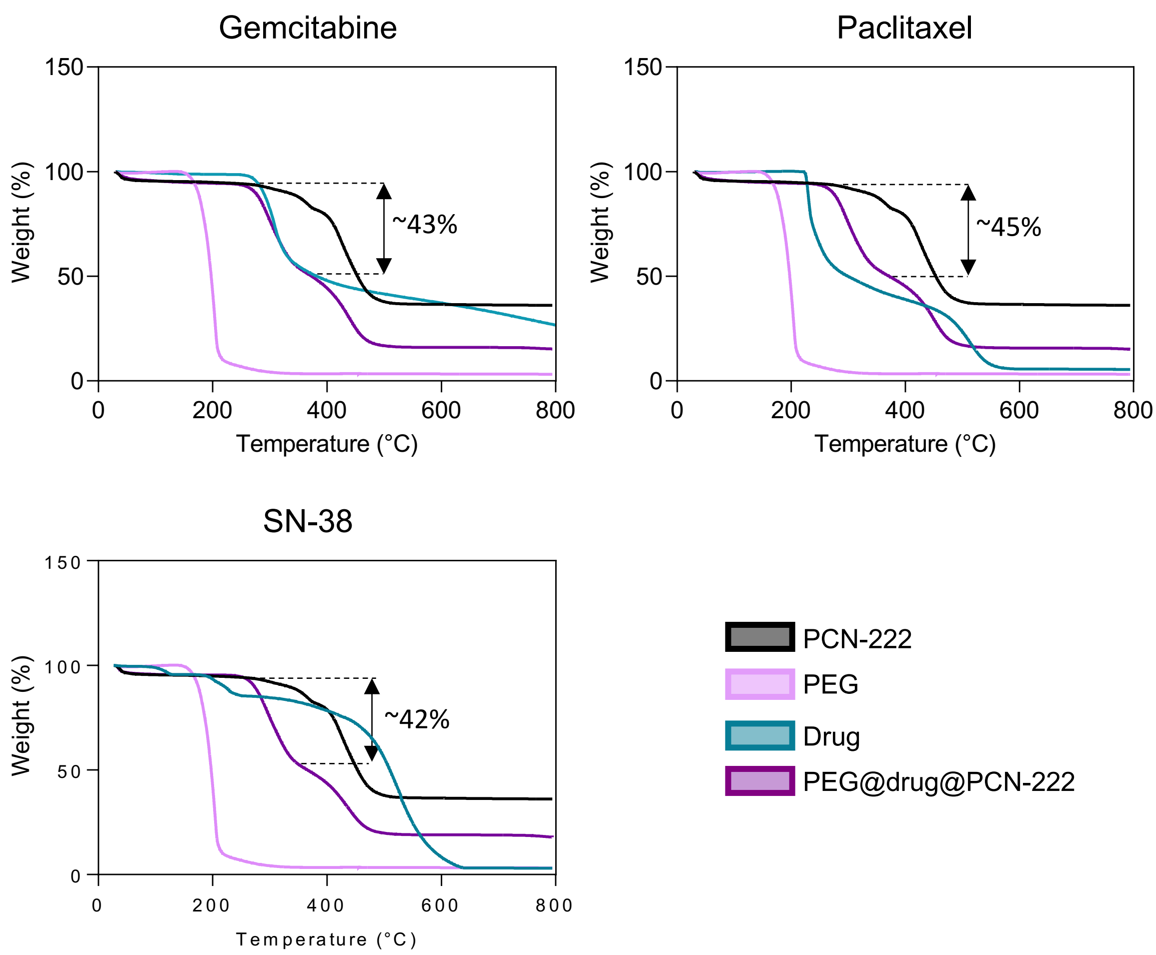

Figure S6. TGA analysis after drug loading and PEGylation of PCN-222. The weight-loss curve of PEG@drug@PCN-222 was compared to the ones of PCN-222, PEG and free drug.

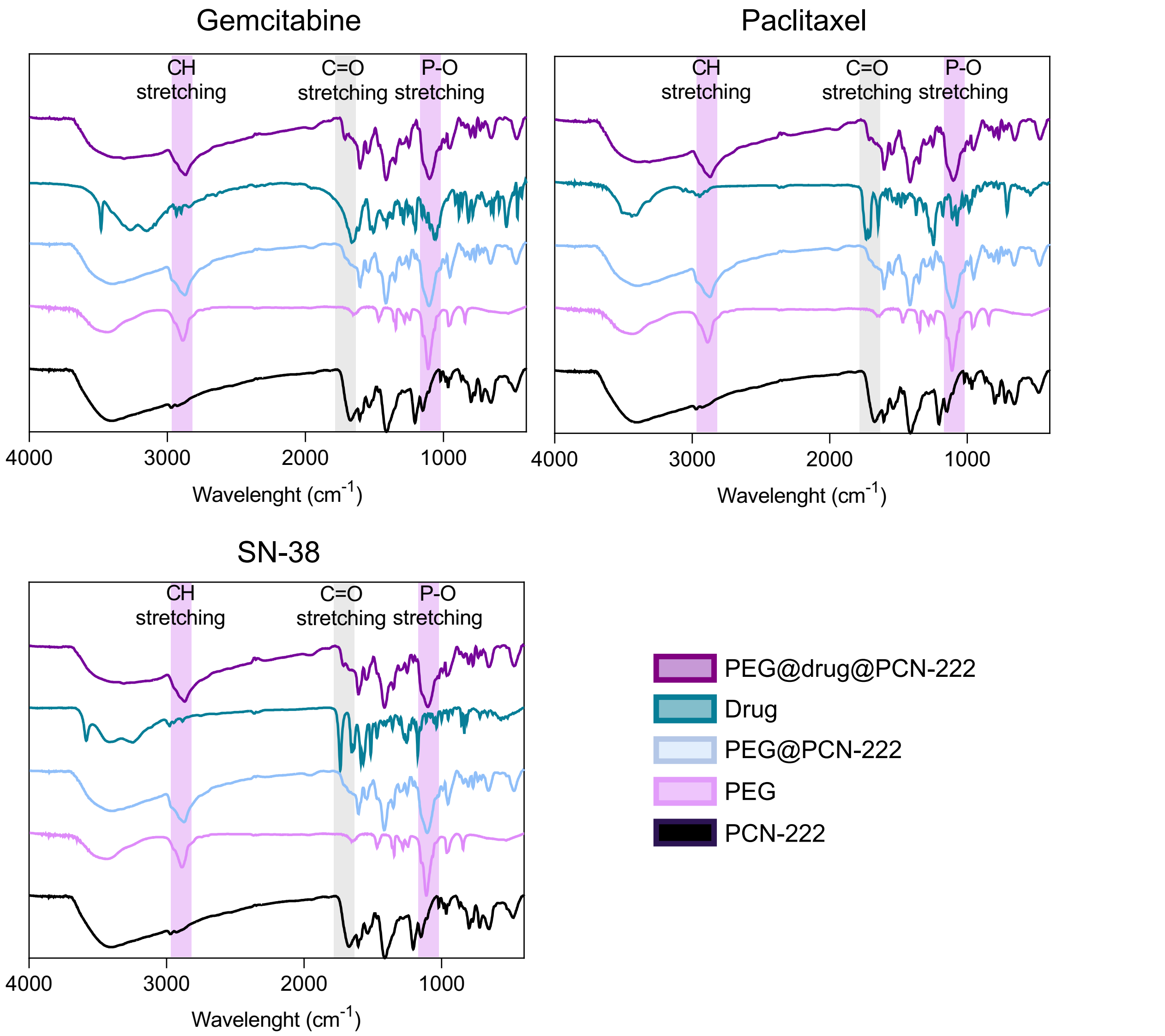

Figure S7. FT-IR spectra of PCN-222 after drug loading and PEGylation. FT-IR spectra were measured for the three drug-loaded PCN-222. Colored rectangles highlight the characteristic peaks of PEG (2,866 and 1,088 cm-1 corresponding to the stretching vibrations of the C-H and P-O) and the three drugs, all sharing the peak at 1,750 cm-1 corresponding to the C=O group.

S4. *In vitro* methods

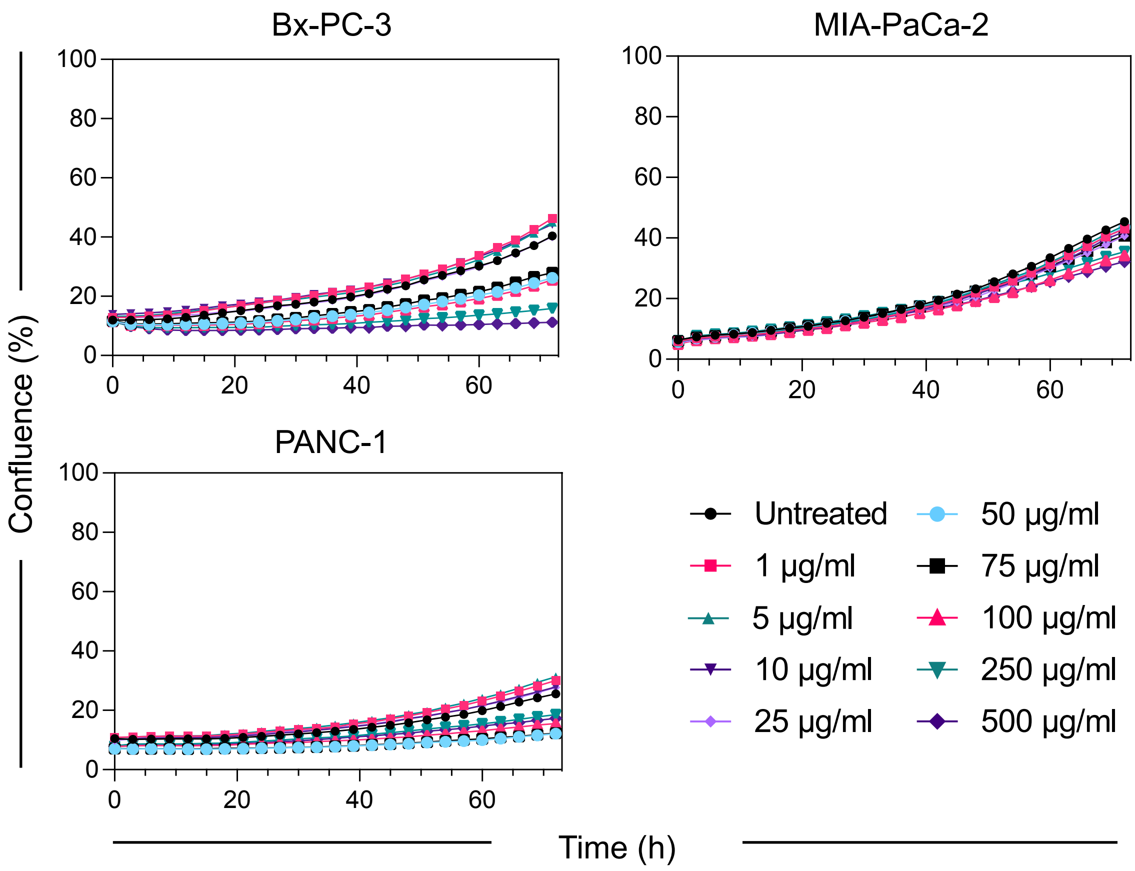

Figure S8. Incucyte live-cell image analysis of cancer cells incubated with PEG@PCN-222. The graphs show the growth curves obtained by the live-cell image analysis of pancreatic cancer cells (Bx-PC-3, MIA-PaCa-2, and PANC-1) at different concentrations for a 72-h incubation period. Data are presented as mean ± SD (n=5).

Table S2. | IC50 values for gemcitabine, paclitaxel, and SN-38 in PDAC cell lines. The IC50 values were calculated for Bx-PC-3, MIA-PaCa-2, and PANC-1 cell lines using both MTS and live-cell imaging system (Incucyte). Values are compared to those obtained from Sanger drug screening data (https://www.cancerrxgene.org/)

		**MTS**	**Incucyte**	**Database**
	Bx-PC-3	0.003	0.03	0.02
Gemcitabine	MIA-PaCa-2	0.009	0.09	0.01
	PANC-1	0.03	-	0.02
	Bx-PC-3	0.004	0.002	0.06
Paclitaxel	MIA-PaCa-2	0.001	0.002	0.04
	PANC-1	0.002	0.004	0.14
	Bx-PC-3	0.0004	0.001	0.13
SN-38	MIA-PaCa-2	0.0008	0.01	0.04
	PANC-1	0.0018	-	0.6

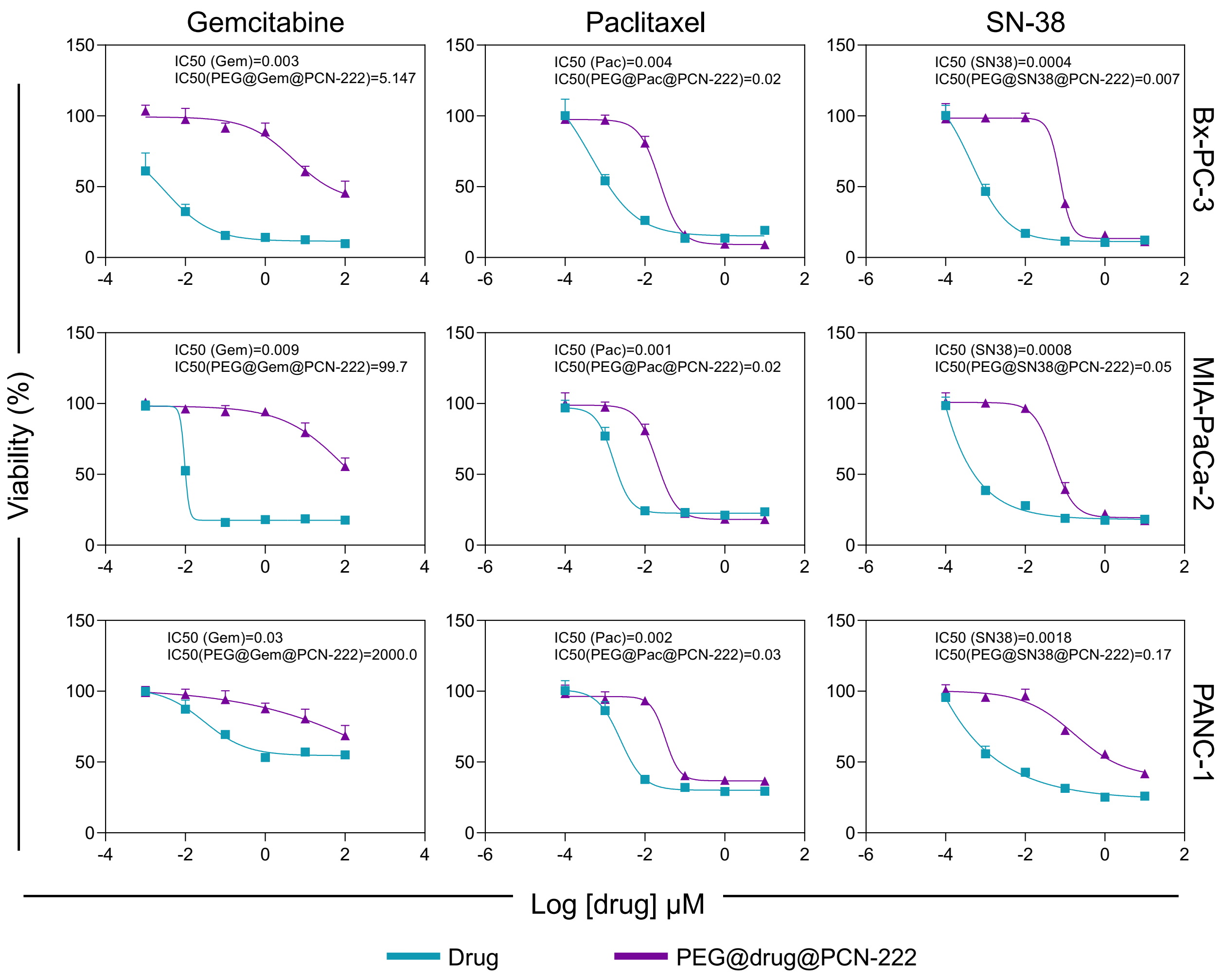

Figure S9. IC50 values of PEG@drug@PCN-222 in PDAC cells. Graphs showing the 50% inhibitory concentrations (IC50) of the PEG@drug@PCN-222 in Bx-PC-3, MIA-PaCa-2, and PANC-1 cell lines calculated using MTS assay. Cells were exposed to the drugs for 72 h. All values were expressed relatively to the cell viability of the untreated cells normalized to 100%. Error bars showed standard deviation between replicates (n=3).

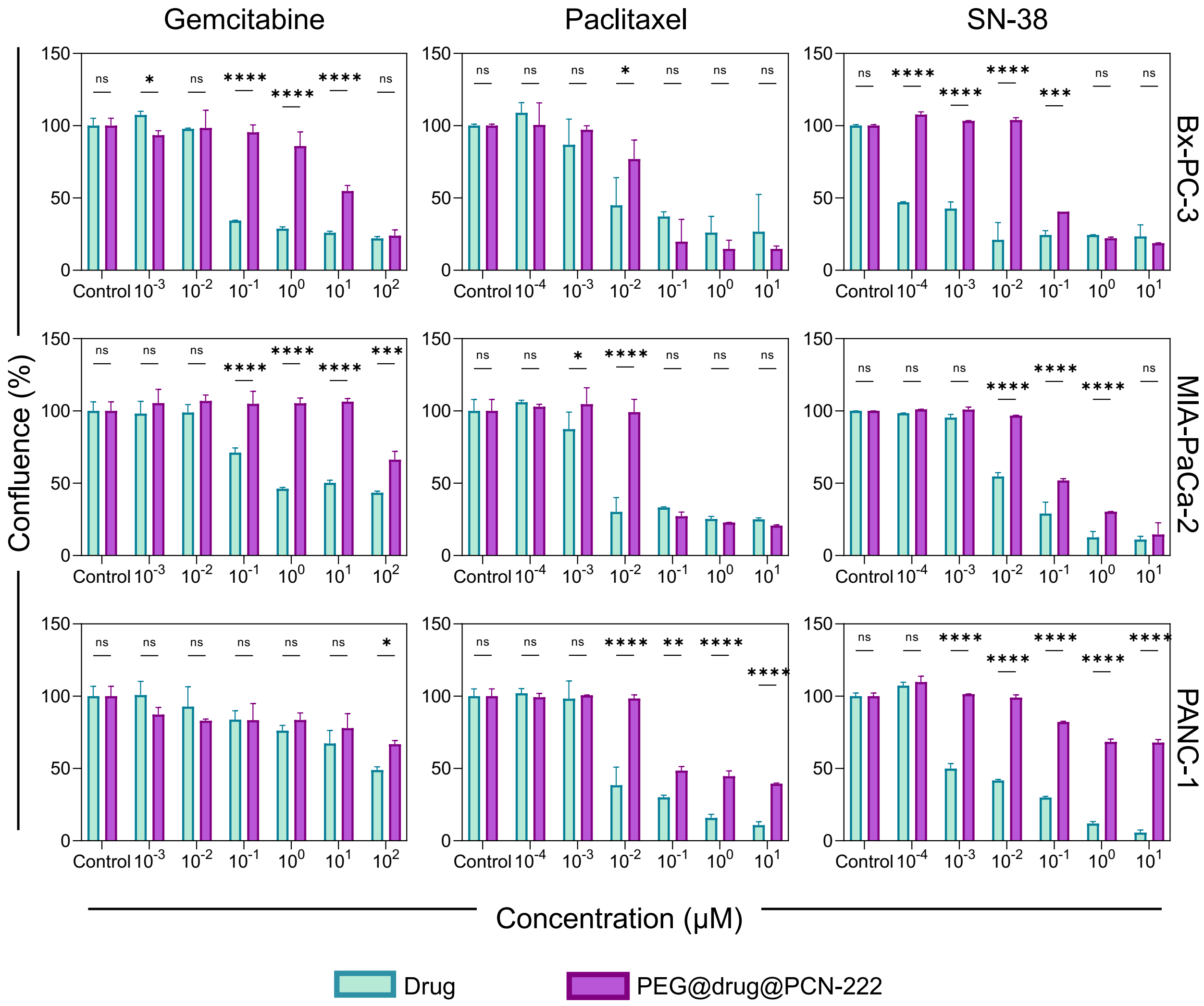

Figure S10. Growth inhibition of PEG@drug@PCN-222 measured using live-cell imaging analysis. Analysis of PEG@Gem@PCN-222, PEG@Pac@PCN-222, and PEG@SN38@PCN-222 at different concentrations after 72h incubation on different PDAC cell lines. The drug-loaded MOF formulations (purple) are compared to the same concentration of free drug (blue) based on the drug loading values. The confluence was normalized by the untreated control (100%). Two-way ANOVA statistical analysis was performed between drug-loaded MOFs and their free drug counterpart at a given concentration, as shown in the graphs. (n=3; ns=non=significant, ****$p<0.00001$, ***$p<0.0001$, **$p<0.01$, *$p<0.05$)

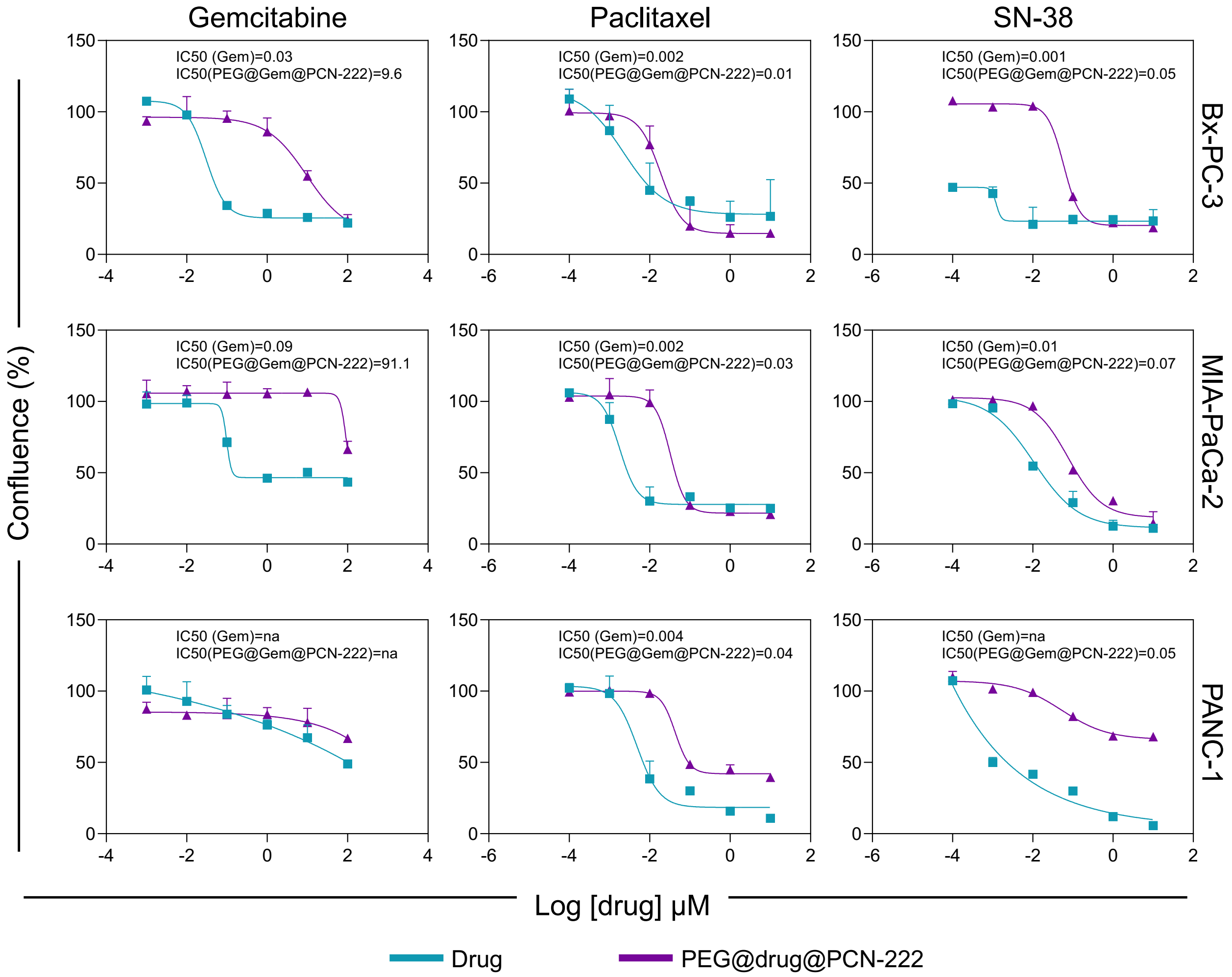

Figure S11. IC50 values of PEG@drug@PCN-222 in PDAC cells measured using live-cell imaging analysis. Graphs showing the 50% inhibitory concentrations (IC50) of the PEG@drug@PCN-222 in Bx-PC-3, MIA-PaCa-2, and PANC-1 cell lines calculated using live-cell imaging analysis. Cells were exposed to the drugs for 72 h. All values were expressed relatively to the cell viability of the untreated cells normalized at 100%. Error bars showed standard deviation between replicates (n=3).

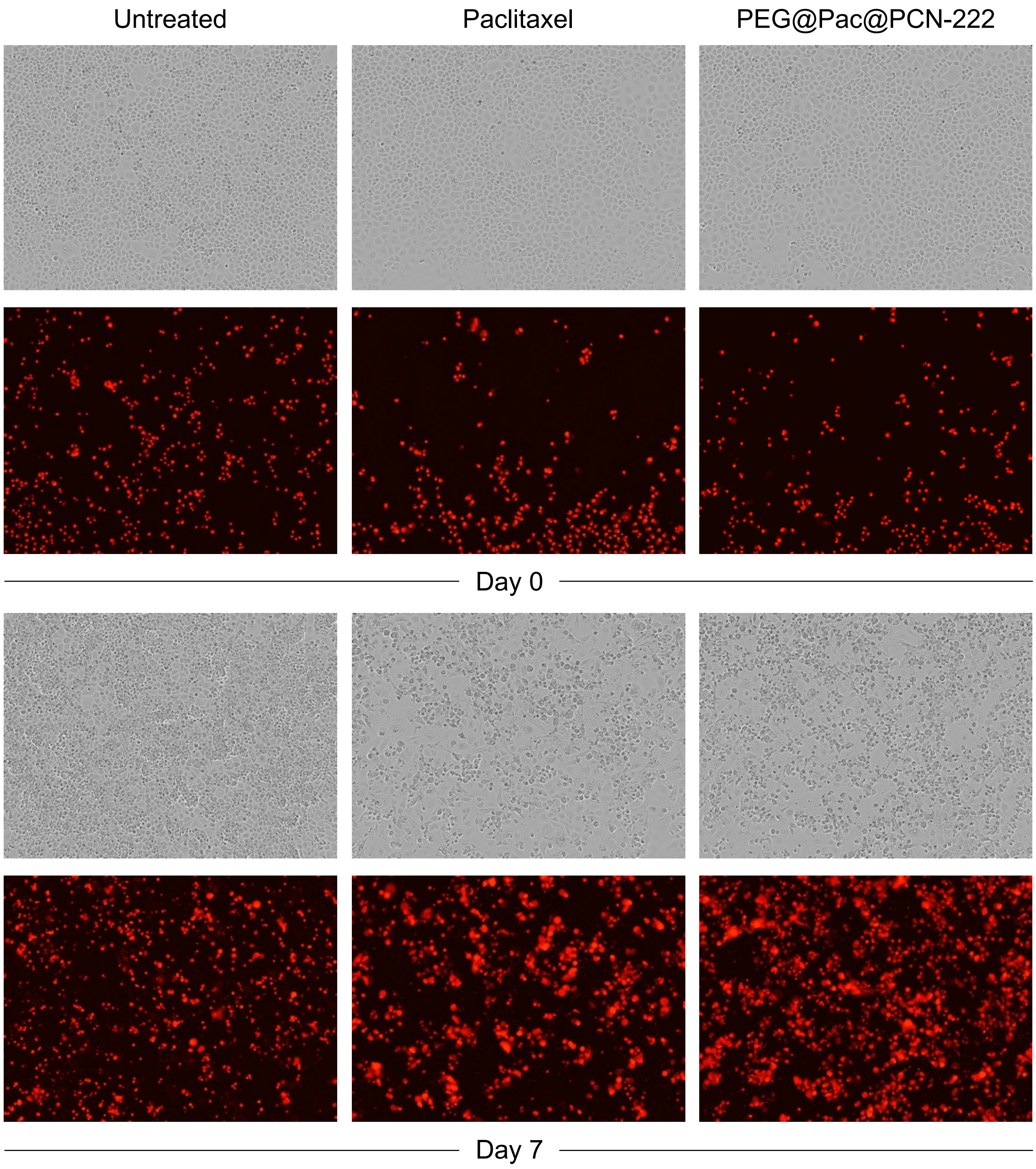

Figure S12. Representative images of PANC-1 cells incubated with paclitaxel and PEG@Pac@PCN-222 (short-term incubation). The cells were incubated with 1 μM of paclitaxel or PEG@Pac@PCN-222 for 6 h, washed two times with media, and then grown in normal conditions for seven days in full media containing CytotoxRed stain. Cells were imaged every hour for seven days.

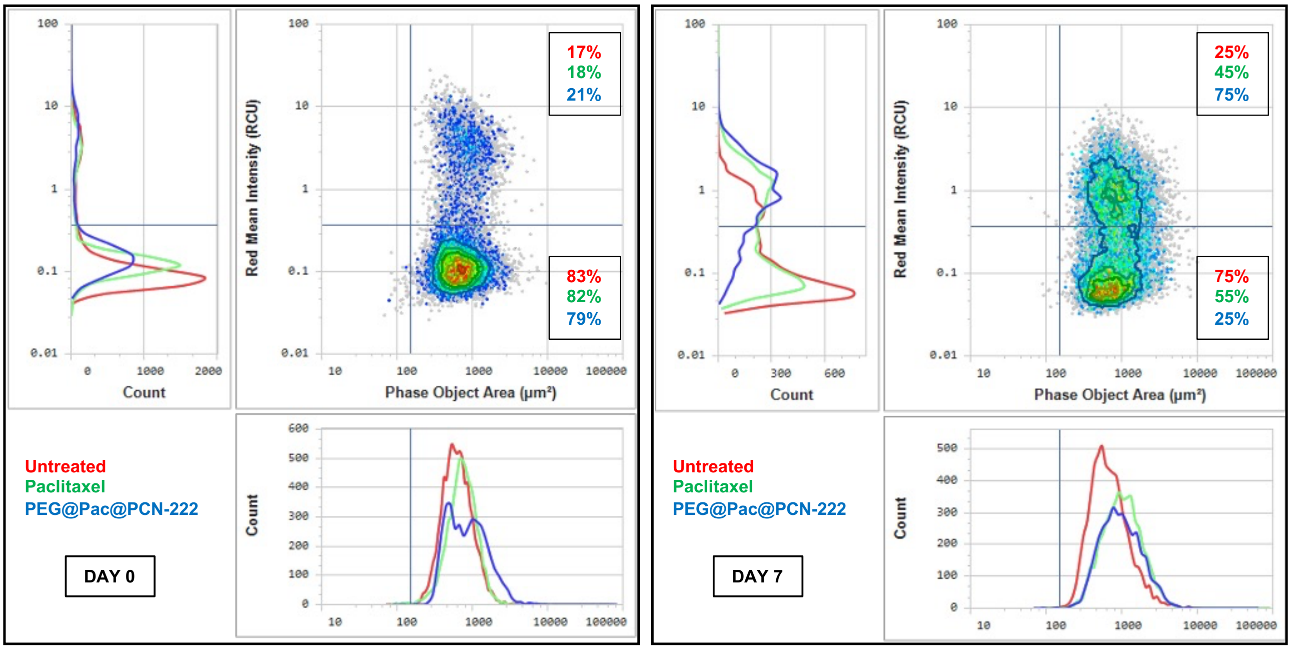

Figure S13. Representative images of Cytotox Red cell imaging analysis. PANC-1 cells incubated with paclitaxel and PEG@Pac@PCN-222 (short-term incubation) and analyzed using Incucyte S3 v2022B software. Classification of the population subset using area, eccentricity, or fluorescence intensity of (dead) cells labeled with Incucyte Cytotox Red dye.

S5. Long-term stability

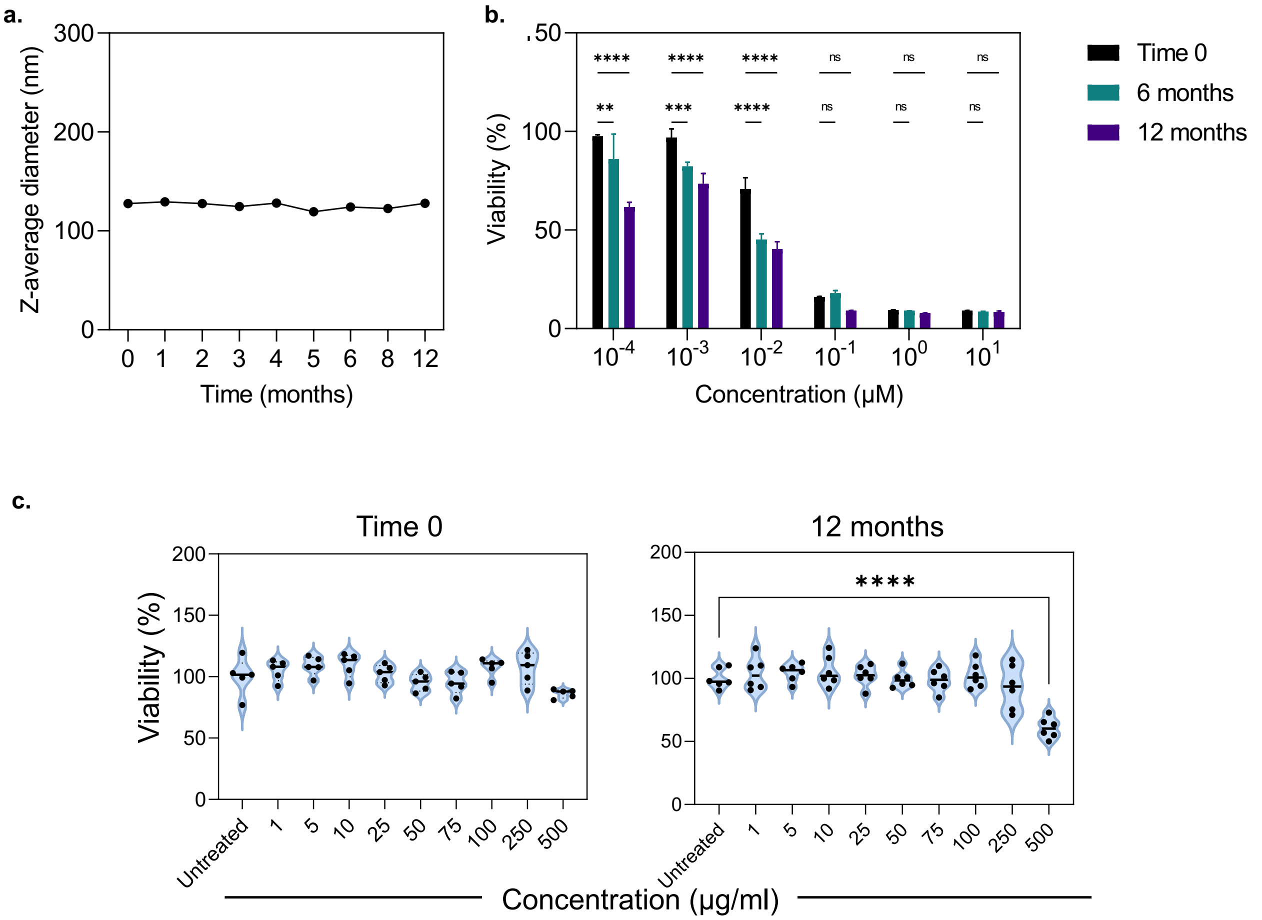

Figure S14. Long-term stability of PCN-222 formulations. a) Long-term stability of PEG@Pac@PCN-222 in water measured using DLS. PEG@Pac@PCN-222 samples were kept in the dark at 4 °C for one year. DLS measurements were taken every month. Data are represented as mean of ± SD (n=3). **b)** Cytotoxicity of PEG@Pac@PCN-222 in BxPC-3 cells after one year. MTS assay of PEG@Pac@PCN-222 at time 0, after 6 and 12 months (storage at 4ºC) at different concentrations after 72 h incubation on BxPC-3 cells. The MOF formulations after 6- and 12-month storage are compared to the same concentration at time 0. **c)** MTS assay of PEG@PCN-222 on BxPC-3 cells after 12 months storage. The biocompatibility of PEG@PCN-222 was evaluated in Bx-PC-3 cells at different concentrations after 72 h incubation. Data are presented as mean ± SD (One-way ANOVA n=3, ****p<0.0001, ***p<0.001, **p<0.01, *p<0.05).

S6. BET area calculation using BETSI[18]

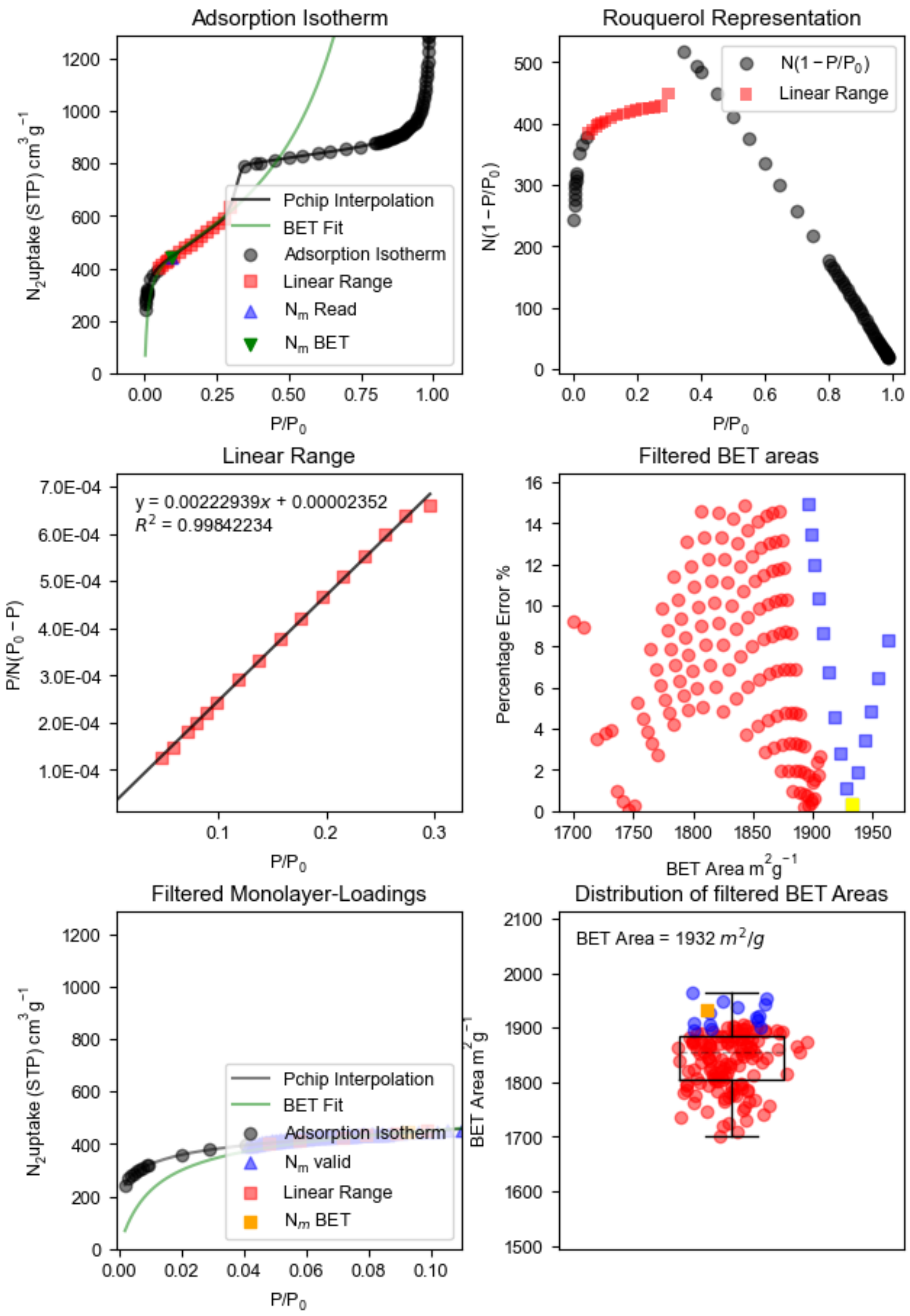

Figure S15. BETSI analysis for PCN-222.

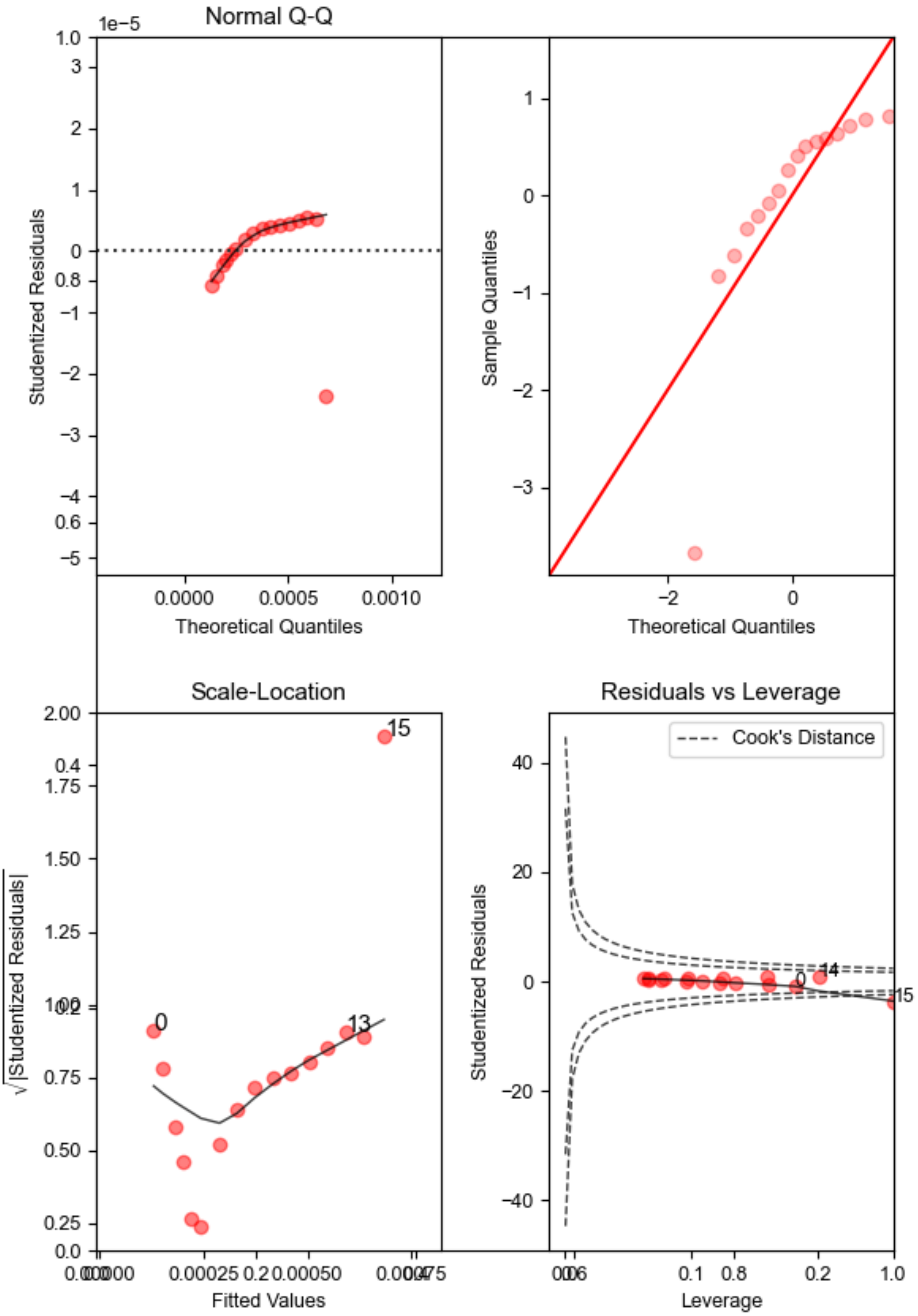

Figure S16. BETSI Regression diagnostics for PCN-222.

S7. *In vivo* studies

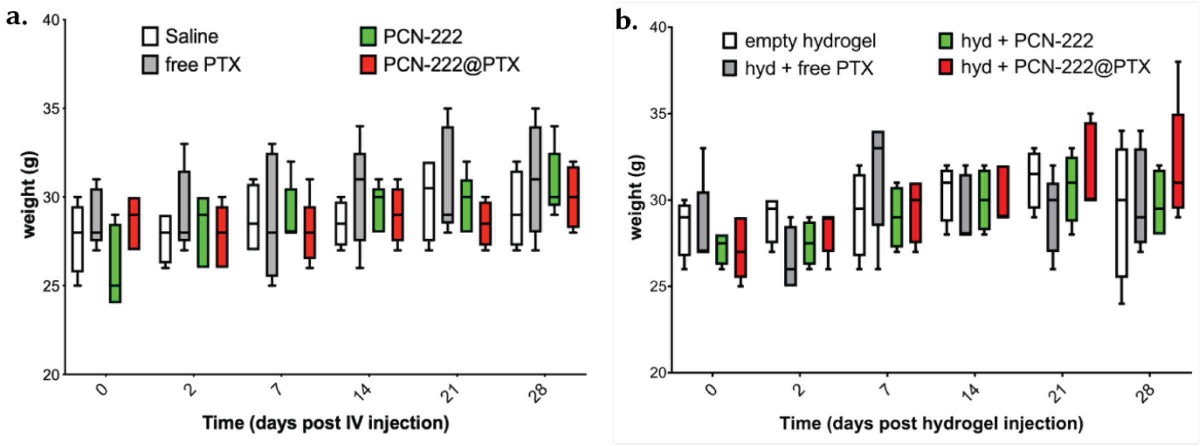

Figure S17. Animal body weight following **a.** intravenous and **b.** hydrogel intraperitoneal administration over a 28-day period (*n=5*).

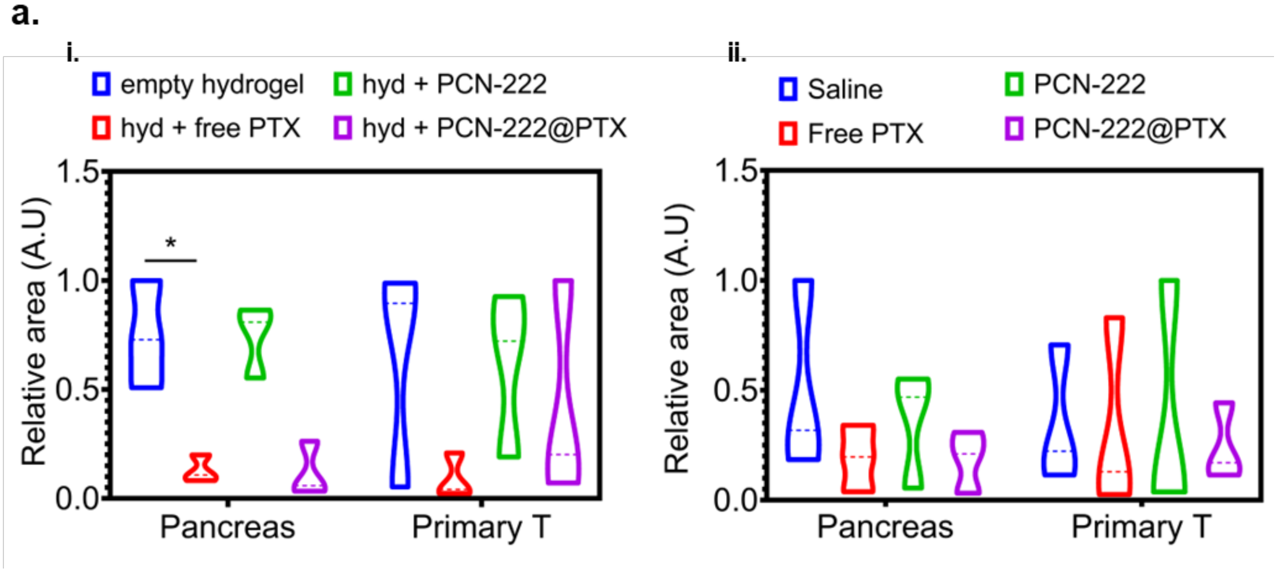

Figure S18. Quantification of the relative area (a.u.) of the pancreas and primary tumors through bioluminescence signal of i) intraperitoneal and (ii) intravenous routes. Statistical analysis was performed using one-way ANOVA with Dunnett's multiple comparison test; *P < 0.05.

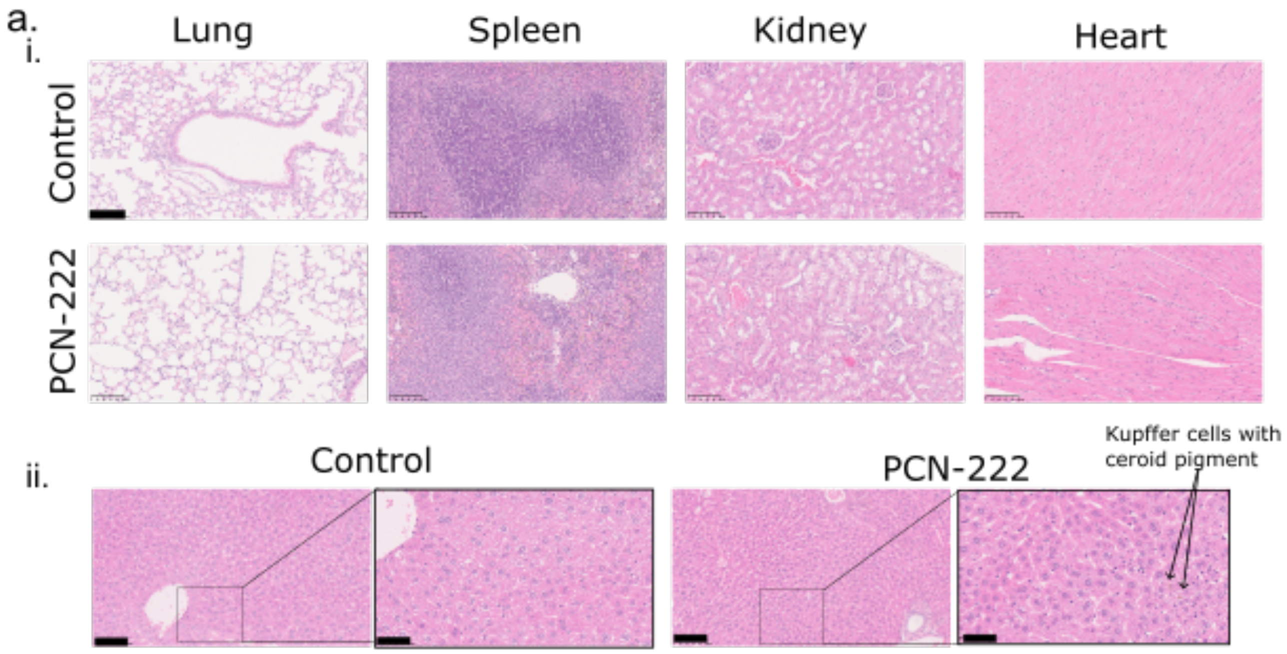

Figure S19. Histopathological images of the collected mouse organs, including **i.** lungs, kidney, heart, spleen (n = 3), and **ii.** liver (n = 3), were stained with hematoxylin and eosin (H&E). Scale bar: 100 μm (i and ii) and 50 μm (ii-zoom-in image).

S8. References

[1] D. Menon, D. Fairen-Jimenez, *Chemrxiv* **2024**, DOI 10.26434/chemrxiv-2024-r43k9-v3.

[2] *Globally Harmonized System of Classification and Labelling of Chemicals (GHS)*, United Nations, **2019**.

[3] P. Z. Moghadam, A. Li, S. B. Wiggin, A. Tao, A. G. P. Maloney, P. A. Wood, S. C. Ward, D. Fairen-Jimenez, *Chemistry of Materials* **2017**, *29*, 2618.

[4] K. M. Jablonka, A. S. Rosen, A. S. Krishnapriyan, B. Smit, *ACS Cent Sci* **2023**, *9*, 563.

[5] T. F. Willems, C. H. Rycroft, M. Kazi, J. C. Meza, M. Haranczyk, *Microporous and Mesoporous Materials* **2012**, *149*, 134.

[6] D. Dubbeldam, S. Calero, D. E. Ellis, R. Q. Snurr, *Mol Simul* **2016**, *42*, 81.

[7] W. L. Jorgensen, D. S. Maxwell, J. Tirado-Rives, *J Am Chem Soc* **1996**, *118*, 11225.

[8] D. S. Wishart, Y. D. Feunang, A. C. Guo, E. J. Lo, A. Marcu, J. R. Grant, T. Sajed, D. Johnson, C. Li, Z. Sayeeda, N. Assempour, I. Iynkkaran, Y. Liu, A. Maciejewski, N. Gale, A. Wilson, L. Chin, R. Cummings, D. Le, A. Pon, C. Knox, M. Wilson, *Nucleic Acids Res* **2018**, *46*, D1074.

[9] Dassault Systèmes Materials Studio 7.0 San Diego: Dassault Systèmes, *BIOVIA* **2013**.

[10] W. L. Jorgensen, J. Tirado-Rives, *Proceedings of the National Academy of Sciences* **2005**, *102*, 6665.

[11] L. S. Dodda, J. Z. Vilseck, J. Tirado-Rives, W. L. Jorgensen, *J Phys Chem B* **2017**, *121*, 3864.

[12] L. S. Dodda, I. Cabeza de Vaca, J. Tirado-Rives, W. L. Jorgensen, *Nucleic Acids Res* **2017**, *45*, W331.

[13] C. E. Wilmer, K. C. Kim, R. Q. Snurr, *J Phys Chem Lett* **2012**, *3*, 2506.

[14] S. L. Mayo, B. D. Olafson, W. A. Goddard, *J Phys Chem* **1990**, *94*, 8897.

[15] A. K. Rappe, C. J. Casewit, K. S. Colwell, W. A. Goddard, W. M. Skiff, *J Am Chem Soc* **1992**, *114*, 10024.

[16] J. C. Boyer, M. P. Manseau, J. I. Murray, F. C. J. M. Van Veggel, *Langmuir* **2010**, *26*, 1157.

[17] X. Chen, Y. Zhuang, N. Rampal, R. Hewitt, G. Divitini, C. A. O'Keefe, X. Liu, D. J. Whitaker, J. W. Wills, R. Jugdaohsingh, J. J. Powell, H. Yu, C. P. Grey, O. A. Scherman, D. Fairen-Jimenez, *J Am Chem Soc* **2021**, *143*, 13557.

[18] J. W. M. Osterrieth, J. Rampersad, D. Madden, N. Rampal, L. Skoric, B. Connolly, M. D. Allendorf, V. Stavila, J. L. Snider, R. Ameloot, J. Marreiros, C. Ania, D. Azevedo, E. Vilarrasa-Garcia, B. F. Santos, X. Bu, Z. Chang, H. Bunzen, N. R. Champness, S. L. Griffin, B. Chen, R. Lin, B. Coasne, S. Cohen, J. C. Moreton, Y. J. Colón, L. Chen, R. Clowes, F. Coudert, Y. Cui, B. Hou, D. M. D'Alessandro, P. W. Doheny, M. Dincă, C. Sun, C. Doonan, M. T. Huxley, J. D. Evans, P. Falcaro, R. Ricco, O. Farha, K. B. Idrees, T. Islamoglu, P. Feng, H. Yang, R. S. Forgan, D. Bara, S. Furukawa, E. Sanchez, J. Gascon, S. Telalović, S. K. Ghosh, S. Mukherjee, M. R. Hill, M. M. Sadiq, P. Horcajada, P. Salcedo-Abraira, K. Kaneko, R. Kukobat, J. Kenvin, S. Keskin, S. Kitagawa, K. Otake, R. P. Lively, S. J. A. DeWitt, P. Llewellyn, B. V. Lotsch, S. T. Emmerling, A. M. Pütz, C. Martí-Gastaldo, N. M. Padial, J. García-Martínez, N. Linares, D. Maspoch, J. A. Suárez del Pino, P. Moghadam, R. Oktavian, R. E. Morris, P. S. Wheatley, J. Navarro, C. Petit, D. Danaci, M. J. Rosseinsky, A. P. Katsoulidis, M. Schröder, X. Han, S. Yang, C. Serre, G. Mouchaham, D. S. Sholl, R. Thyagarajan, D. Siderius, R. Q. Snurr, R. B. Goncalves, S. Telfer, S. J. Lee, V. P. Ting, J. L. Rowlandson, T. Uemura, T. Iiyuka, M. A. van der Veen, D. Rega, V. Van Speybroeck, S. M. J. Rogge, A. Lamaire, K. S. Walton, L. W. Bingel, S. Wuttke, J. Andreo, O. Yaghi, B. Zhang, C. T. Yavuz, T. S. Nguyen, F. Zamora, C. Montoro, H. Zhou, A. Kirchon, D. Fairen-Jimenez, *Advanced Materials* **2022**, *34*, DOI 10.1002/adma.202201502.
